# Prevalence of pneumonia and its associated factors among under-five children in East Africa: a systematic review and meta-analysis

**DOI:** 10.1186/s12887-020-02083-z

**Published:** 2020-05-27

**Authors:** Biruk Beletew, Melaku Bimerew, Ayelign Mengesha, Mesfin Wudu, Molla Azmeraw

**Affiliations:** Department of Nursing, College of Health Sciences, Woldia University, P.O.Box 400, Woldia, Ethiopia

**Keywords:** Pneumonia, Eastern-Africa, Under five children, Indicator Cluster Surveys (MICS) Child Health/Pneumonia.2017

## Abstract

**Background:**

Pneumonia is defined as an acute inflammation of the Lungs’ parenchymal structure. It is a major public health problem and the leading cause of morbidity and mortality in under-five children especially in developing countries. In 2015, it was estimated that about 102 million cases of pneumonia occurred in under-five children, of which 0.7 million were end up with death. Different primary studies in Eastern Africa showed the burden of pneumonia. However, inconsistency among those studies was seen and no review has been conducted to report the amalgamated magnitude and associated factors. Therefore, this review aimed to estimate the national prevalence and associated factors of pneumonia in Eastern Africa

**Methods:**

Using PRISMA guideline, we systematically reviewed and meta-analyzed studies that examined the prevalence and associated factors of pneumonia from PubMed, Cochrane library, and Google Scholar. Heterogeneity across the studies was evaluated using the Q and the I^2^ test. A weighted inverse variance random-effects model was applied to estimate the national prevalence and the effect size of associated factors. The subgroup analysis was conducted by country, study design, and year of publication. A funnel plot and Egger’s regression test were used to see publication bias. Sensitivity analysis was also done to identify the impact of studies.

**Result:**

A total of 34 studies with 87, 984 participants were used for analysis. The pooled prevalence of pneumonia in East Africa was 34% (95% CI; 23.80–44.21). Use of wood as fuel source (AOR = 1.53; 95% CI:1.30–1.77; I^2^ = 0.0%;*P* = 0.465), cook food in living room (AOR = 1.47;95% CI:1.16–1.79; I^2^ = 0.0%;*P* = 0.58), caring of a child on mother during cooking (AOR = 3.26; 95% CI:1.80–4.72; I^2^ = 22.5%;*P* = 0.26), Being unvaccinated (AOR = 2.41; 95% CI:2.00–2.81; I^2^ = 51.4%;*P* = 0.055), Child history of Acute Respiratory Tract Infection (ARTI) (AOR = 2.62; 95% CI:1.68–3.56; I^2^ = 11.7%;*P* = 0.337) were identified factors of pneumonia.

**Conclusion:**

The prevalence of pneumonia in Eastern Africa remains high. This review will help policy-makers and program officers to design pneumonia preventive interventions.

## Background

Pneumonia is defined as an acute inflammation of the Lungs’ parenchymal structure. It can be classified based on place of acquisition: as community acquired or hospital acquired; based on its causative agents/ mechanism as bacterial, viral, fungal, Aspiration, or ventilator-associated pneumonia; based on the anatomy of the lungs involved as lobar pneumonia, bronchial pneumonia or acute interstitial pneumonia; and on the basis of its clinical severity as “no pneumonia”, “pneumonia” or “severe pneumonia” [1–3].

Under-five children are more vulnerable to pneumonia and pneumonia remains the leading cause of morbidity and mortality in those children [4]. According to a global estimate made in 2000, approximately 156 million cases of pneumonia had occurred each year in under-five children, of which 151 million episodes were in the developing countries and about 1.2 million of them were end up in death. South-east Asia and Africa were the two continents with high magnitude of childhood pneumonia, having an estimated of 61 million and 35 million annual cases of pneumonia in under-five children respectively [5]. The magnitude of under-five pneumonia was decreased to 120 million (with 0.88 million deaths) in 2010 and to 102 million (with 0.7 million deaths) in 2015 globally. These decrement was due to decrease in the magnitude of its key risk factors, increasing socioeconomic development and preventive interventions, improved access to care, and quality of care in hospitals. Despite this progress, pneumonia is still a major public health problem for children especially in developing countries [4].

Globally, many researches had been conducted to identify risk factors of pneumonia. Despite the inconsistency of findings, low birth weight, malnutrition, indoor air pollution, parental smoking, being unvaccinated, overcrowding, lack of separate kitchen, being not on exclusive breast feeding, and maternal education were identified as factors associated with occurrence of pneumonia in under-five children [6–9].

Besides, in East African countries different researchers had tried to investigate the magnitude of pneumonia in under-five children and have reported a prevalence ranges from 5.5% [10] up to 89.8% [11]. They had also identified risk factors for pneumonia among under-five children. But, reported finding lack consistency and as per the investigators knowledge there is no a systematic review and meta-analysis conducted to address these inconsistent findings reported from East African countries. Moreover, assessing the magnitude of pneumonia and identifying its associated factors for risk based diagnosis of pneumonia contribute in better interventions and helps to reduce the higher burden of pneumonia in under-five children. Hence, this systematic review and meta-analysis was conducted to assess the magnitude of pneumonia and its associated factors among under-five children in East Africa.

## Methods

### Reporting

The results of this review were reported based on the Preferred Reporting Items for Systematic Review and Meta-Analysis statement (PRISMA) guideline (Supplementary file-PRISMA checklist) and, it is registered in the Prospero database: (PROSPERO 2019: CRD42019136707) Available from https://www.crd.york.ac.uk/PROSPERO/#myprosperoID = CRD42019136707.

### Searching strategy and information sources

We identified studies providing data on the prevalence of and potential risk factors of pneumonia among under-five children, with the search focused on Eastern Africa from PubMed, Cochrane library, and Google Scholar. The search included MeSH terms and keywords, combinations, and snowball searching in references list of papers found through the data base search to retrieve additional articles. Articles with incomplete reported data were handled through contacting corresponding authors. Unpublished studies were retrieved from the official websites of international and local organizations and universities. The search was performed by keywords, medical subject headings (MeSH) terms. We used the search terms independently and/or in combination using “OR” or “AND”. The core search terms and phrases were “under five”, “children”, “child”, “infant”, and “pneumonia”, “respiratory infection”, causes, risk factors, determinants, associated factors, predictors and Eastern Africa. The search strategies were developed using different Boolean operators. Remarkably, to fit advanced PubMed database, the following search strategy was applied: (prevalence OR magnitude OR epidemiology) AND (causes OR determinants OR associated factors OR predictors OR risk factors) AND (children [MeSH Terms] OR under five OR child OR childhood) AND (pneumonia [MeSH Terms] OR respiratory tract infection) AND Eastern Africa. We also screened at the reference lists of the remaining papers to identify additional relevant studies to this review.

### Study selection / eligibility criteria

Retrieved studies were exported to reference manager software, Endnote version 8 to remove duplicate studies. Two investigators (BB and AM) independently screened the selected studies using their titles and abstracts before retrieval of full-text papers. We used pre-specified inclusion criteria to further screen the full-text articles. Disagreements were discussed during a consensus meeting with other reviewers (MW and MB) for the final selection of studies to be included in the systematic review and meta-analysis.

### Inclusion and exclusion criteria

All observational studies (cross-sectional, case-control, and cohort studies) were included. Those studies had reported the prevalence and/or at least one associated factors of pneumonia among under-five children and published in English language from 2000 up to 2019 in Eastern Africa were considered. A consideration was extended to unpublished work among children under five were also considered. Citations without abstract and/or full-text, anonymous reports, editorials, and qualitative studies were excluded from the analysis. Furthermore, researches which did not report our results of interest were excluded. Regarding inclusion and exclusion criteria of included studies, children below 59 months of age with mother / care giver visiting out patients department during data collection period were included. Severely sick child need life treating intervention and whose mother / care givers refused were excluded from the study.

### Quality assessment

Duplicate articles were removed using Endnote (version X8) after combining the Database search results. The Joanna Briggs Institute (JBI) quality appraisal checklist was used [12, 13]*.* Four independent authors appraised the quality of the studies. The appraisal was repeated by exchanging with each other. Thus, one paper was appraised by two Authors. Any disagreement between the reviewers was solved by taking the mean score of the two reviewers. Studies were considered as low risk or good quality when it scored 5 and above for all designs (cross sectional, case control, and cohort) and were included [12, 13] whereas the score was 4 and below the studies considered as high risk or poor quality and was not included.

### Data extraction

The authors developed data extraction form on the excel sheet which includes author name, year of publication, study country, study design, sample size, prevalence of pneumonia, and categories of factors reported. The data extraction sheet was piloted using 4 papers randomly. The extraction form was adjusted after piloted the template. Two of the authors extracted the data using the extraction form in collaboration. The third and fourth authors check the correctness of the data independently. Any disagreements between reviewers were resolved through discussions with a third reviewer and fourth reviewer if required. The mistyping of data was resolved through crosschecking with the included papers. If we got incomplete data, we excluded the study after two attempts were made to contact the corresponding author by email.

### Outcome measurement

Pneumonia was considered when under five children with cough and/or difficulty of breathing, have fast breathing and/or chest indrawing and suggestive X-ray findings [14, 15].

### Statistical analysis

After the data was extracted using Microsoft Excel format we imported the data to STATA version 14.0 statistical software for further analysis. Using the binomial distribution formula, Standard error was calculated for each study. We pooled the overall magnitude estimates of pneumonia by a random effect meta-analysis [16]. The pooled prevalence of pneumonia with 95% CI was presented using forest plots and Odds ratio (OR) with 95% CI was also presented in forest plot to show the associated factors of pneumonia. We examined the heterogeneity between the studies using Cochrane’s Q statistics (Chi-square), invers variance (I2) and *p*-values [17].

In this study, the I^2^ statistic value of zero indicates true homogeneity, whereas the value 25, 50, and 75% represented low, moderate and high heterogeneity respectively [18, 19]. For the data identified as heterogeneous, we conducted our analysis by random-effects model analysis. In addition subgroup analysis was done by the study country, design, and year of publication. When statistical pooling is not possible, non-pooled data was presented in table form. Sensitivity analysis was employed to see the effect of a single study on the overall estimation. Publication bias was checked by funnel plot and more objectively through Egger’s regression test [20].

## Result

### Study selection

**Fig. 1:**
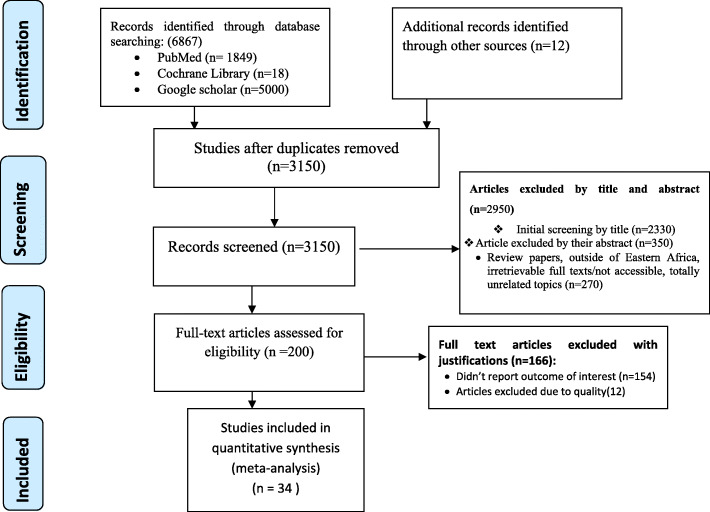
PRISMA flow diagram showed the results of the search and reasons for exclusion

A total of 6879 studies were identified using electronic searches (through Databases searching (*n* = 6867)) and other sources (*n* = 12)) that were conducted from 2000 up to 2019. After duplication removed, a total of 3150 articles remained (3729 duplicated). Finally, 200 studies were screened for full-text review and, 34 articles with (*n* = 87,984 patients) were selected for the prevalence and/ or associated factors analysis (Fig.1).

### Characteristics of included studies

Table 1 summarizes the characteristics of the 34 included studies in the systematic review and meta-analysis [10, 11, 21–37, 39–52]. 16 studies were found in Ethiopia [10, 22–36], 8 in Kenya [11, 37, 39–43], 2 in Uganda [51, 52],1 Eritrea [21], 1 in Somali [44],4 Sudan [45–48],2 Tanzania [49, 50].

**Table 1:** Distribution of studies on the prevalence and determinants of pneumonia among under five children in East Africa, 2000–2019

Author/Reference	Year	Country	Study design	Sample size	Prevalence (%)	Quality score
Shah et al [21]	2012	Eritrea	Cross-sectional	1502	29	5/8
Negash et al [22]	2019	Ethiopia	Cohort	362	21.5	9/11
Abaye et al [23]	2019	Ethiopia	Cross-sectional	477	18.4	6/8
Lema et al [24]	2019	Ethiopia	Cross-sectional	344	17.7	7/8
Fekadu et al [25]	2014	Ethiopia	Cross-sectional	286	16.1	7/8
Dadi et al [26]	2014	Ethiopia	Case control	356		7/8
Geleta et al [27]	2016	Ethiopia	case control	382		8/8
Shibre et al [10]	2015	Ethiopia	Cross-sectional	458	5.5	6/8
Tegenu et al [28]	2018	Ethiopia	Cross-sectional	306	28.1	5/8
Abuka et al [29]	2017	Ethiopia	Cross-sectional	206	33.5	7/8
Workineh et al [30]	2017	Ethiopia	Case control	558		7/10
Markos et al [31]	2019	Ethiopia	Case control	435		7/10
Gedefaw et al [32]	2015	Ethiopia	Case control	244		8/10
Tadesse et al [33]	2015	Ethiopia	Cross-sectional	150	26.7	8/8
Adhanom et al [34]	2019	Ethiopia	Cross-sectional	252	43.7	5/8
Lenda et al [35]	2018	Ethiopia	Cross-sectional	458	17.6	8/8
Deribew et al [36]	2007	Ethiopia	case control	168	22.6	9/10
MANYA et al [37]	2005	Kenya	case control	188		7/10
Keter et al [38]	2015	Kenya	Cross-sectional	422	67.1	6/8
Onyango et al [39]	2012	Kenya	case control	206		7/10
Muthumbi et al [40]	2017	Kenya	Cross-sectional	1483		7/8
Ndungu et al [41]	2018	Kenya	Cross-sectional	323	74.3	6/8
Walekhwa et al [42]	2019	Kenya	Cross-sectional	206	20.39	7/8
Sikolia et al [43]	2002	Kenya	Cross-sectional	300	69.7	6/8
Ásbjörnsdóttir et al [11]	2012	Kenya	Cohort	365	89.8	10/11
Kinyoki et al [44]	2017	Somalia	Cross-sectional	73,778	17	6/8
Gritly et al [45]	2018	Sudan	Cross-sectional	40	65	7/8
Salih et al [46]	2014	Sudan	Cross-sectional	195	10.32	5/8
Gabbad et al [47]	2014	Sudan	Cross-sectional	282	20.2	7/8
Deng et al [48]	2019	Sudan	case control	108		8/10
Ndosa et al [49]	2015	Tanzania	Cross-sectional	12.3		5/8
Lugangira et al [50]	2017	Tanzania	Cross-sectional	1130	22	8/8
Lindstrand et al [51]	2018	Uganda	Cross-sectional	1723	56	6/8
Tuhebwe et al [52]	2014	Uganda	Cross-sectional	278	9.4	7/8

23 studies were cross-sectional, while the others used either case-control (*n* = 9) or cohort (*n* = 2) study design. Most of the studies 23/34(70.5%) were published between 2015 and 2019. The studies included participants, ranging from 40 [45] to 73,778 [44] (Table 1).

### Meta-analysis

#### Prevalence of pneumonia among fewer than five children in Ethiopia

**Fig. 2:**
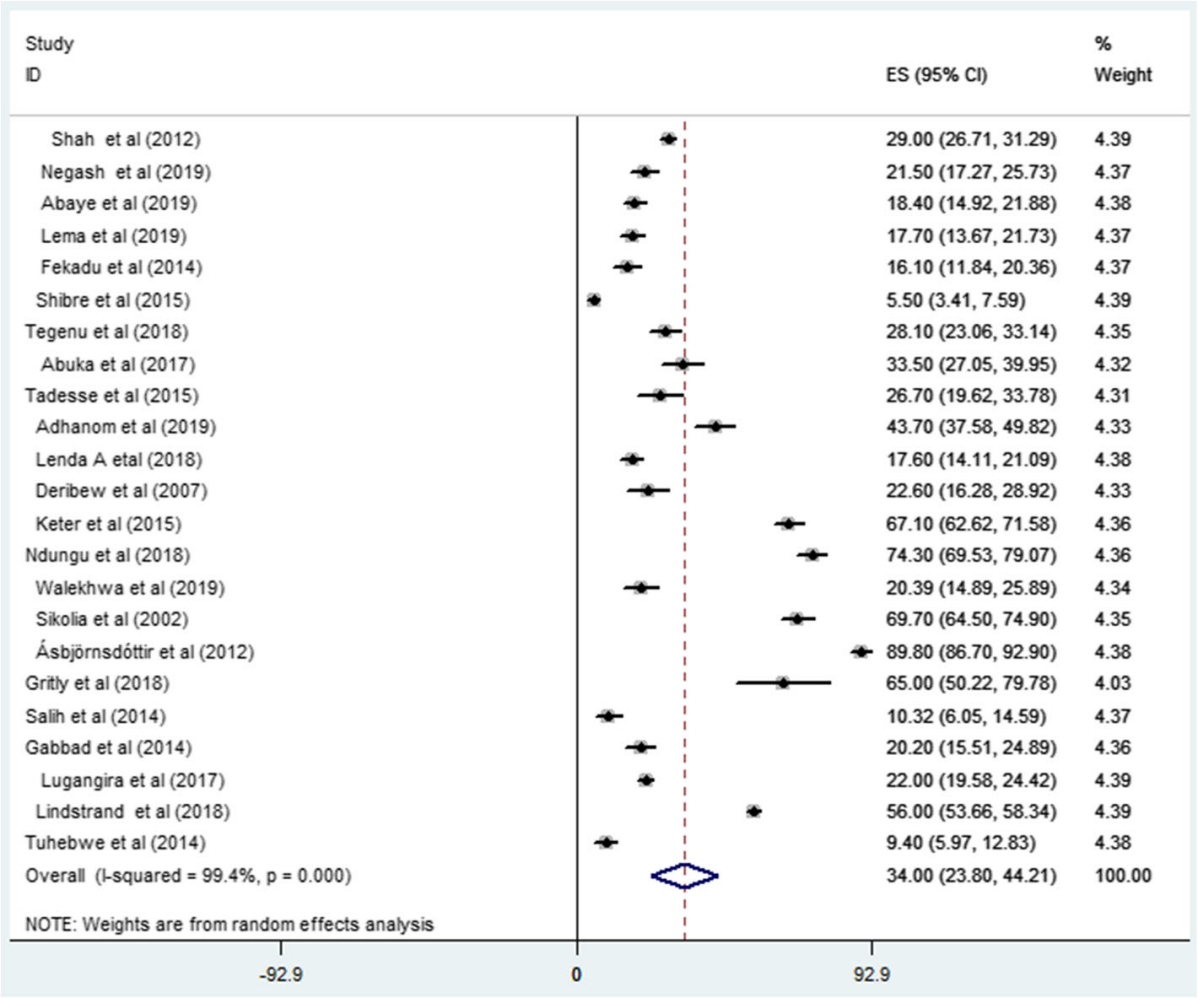
Forest plot showing the pooled prevalence of pneumonia among under-five children in Eastern Africa from 2000 up to 2019

Most of the studies (*n* = 23) had reported the prevalence of pneumonia [10, 11, 21–25, 28, 29, 33–36, 41–47, 50–52]. The prevalence of pneumonia were ranged from 5.5% [10] up to 89.8% [11]. The random-effects model analysis from those studies revealed that, the pooled prevalence of pneumonia in East Africa was found to be 34% (95%CI; 23.80–44.21; I^2^ = 99.4%; *p* < 0.001) (Fig.2).

#### Subgroup analysis of the prevalence of pneumonia in eastern Africa

The subgroup analysis was done through stratified by country, study design, and year of publication. Based on this, the prevalence of pneumonia among under five children was found to be 29 in Eritrea, 22.62 in Ethiopia, 64.3 in Kenya, 29.71 in Sudan, 22 in Tanzania, and 32.72 in Uganda (Supplementary Fig. 1 and Table 2). Based on the study design, the prevalence of pneumonia was found to be 32.33 in cross-sectional studies, 55.68% in cohort studies and 22.6 in case control studies (Supplementary Fig. 2 and Table 2). Based on the year of publication, the prevalence of pneumonia was found to be 33.4 from 2000 to 2015, while it was 34.29 from studies conducted from 2016 to 2019(Supplementary Fig. 3, Table 2).

**Table 2:** Subgroup analysis of the prevalence of pneumonia in Eastern Africa by country, design and year of publication

Variables	Characteristics	Pooled prevalence (95% CI)	I^2^(P-value)
By country	Eritrea	29.00(26.71–31.29)	–
	Ethiopia	22.62(16.37–28.87)	96%(< 0.001)
	Kenya	64.31(42.70–85.92)	99.1%(< 0.001)
	Sudan	29.71(11.83–47.60)	96.1%(< 0.001)
	Tanzania	22.00(19.58–24.42)	–
	Uganda	32.72(12.95–78.38)	99.8%(< 0.001)
By design	Cross-sectional	32.33(23.22–41.44)	99.2% (< 0.001)
	Cohort	55.68(−11.27–122.60)	99.8%(< 0.001)
	Case control	22.60(16.28–28.92)	–
By year of publication	2000–2015	33.40(11.54–55.25)	99.6% (< 0.001)
	2016–2019	34.29(23.05–44.21)	99.2%(< 0.001)

### Sensitivity analysis

We employed a leave-one-out sensitivity analysis to identify the potential source of heterogeneity in the analysis of the prevalence of pneumonia in Eastern Africa. The results of this sensitivity analysis showed that our findings were not dependent on a single study. Our pooled estimated prevalence of pneumonia varied between 31.38(22.93–39.83) [11] and 35.3(25.13–45.49) [10] after deletion of a single study (Supplementary Fig. 4).

### Publication Bias

We have also checked publication bias and a funnel plot showed symmetrical distribution. Egger’s regression test *p*-value was 0.63, which indicated the absence of publication bias (Supplementary Fig. 5).

### Factors associated with pneumonia

Out of the total included studies 18 studies [10, 22–28, 30–35, 37, 39, 40, 43] revealed the factors associated with pneumonia among under five children in Eastern Africa (Table 3).

**Table 3:** Factors associated with pneumonia in East Africa

Variables	Odds ratio(95%CI)	Author (reference)	Year	Pooled AOR(95%CI)	I^2^(P-value)
Use of wood as fuel source	1.15(0.47,1.88)	Negash et al [22]	2019	1.53(1.30, 1.77)	0.0% (0.465)
	2.1 (0.58,6.98)	Lema et al [24]	2019		
	7.41 (2.75,19.95)	Fekadu et al [25]	2014		
	1.49 (0.32,6.36)	Shibre et al [10]	2015		
	3.41(1.5,7.7)	Tegenu et al [28]	2018		
	2.92 (0.78,10.84)	Abuka et al [29]	2017		
	1.78(0.28,1.09)	Onyango et al [39]	2012		
	1.42(0.28,0.92)	Sikolia et al [43]	2002		
Cook food in living room	2.12(0.76, 5.92)	Lema et al [24]	2019	1.47(1.16–1.79)	0.0% (0.58)
	1.5(1.42, 5.4)	Dadi et al [26]	2014		
	2.1(1.2, 3.7)	Geleta et al [27]	2016		
	3.27(1.4,7.9)	Tegenu et al [28]	2018		
	2.16(1.17,3.99	Lenda et al [35]	2018		
	1.35(0.3,0.99)	Sikolia et al [43]	2002		
Caring of a child on mother during cooking	11.76(4.6,30.08)	Lema et al [24]	2019	3.26(1.80–4.72)	22.5% (0.26)
	5.38(2.13,9.65)	Fekadu et al [25]	2014		
	1.7(1.317,7.362)	Dadi et al [26]	2014		
	2.55(1.33,6.5)	Tegenu et al [28]	2018		
	1.37(0.24,7.83)	Abuka et al [29]	2017		
	7.37(2.55,21.32)	Tadesse et al [33]	2015		
	6.2(3.25,11.83)	Lenda et al [35]	2018		
Being unvaccinated	2.6(0.8, 8.1)	Negash et al [22]	2019	2.41(2.00–2.81)	51.4% (0.055)
	1.6(0.9,2.9)	Geleta et al [27]	2016		
	4.62(2.64,11)	Tegenu et al [28]	2018		
	1.68(0.16,2.42)	Abuka et al [29]	2017		
	2.77(0.19,0.54)	Workineh et al [30]	2017		
	2.67(0.15,0.92)	MANYA et al [37]	2005		
	1.68(0.16,2.42)	Onyango et al [39]	2012		
Non-exclusive breast feeding	1.51(0.88,2.58)	Negash et al [22]	2019	2.47(1.79, 3.16)	65.0% (0.01)
	6(3.33,10.8)	Abaye et al [23]	2019		
	2.49(0.05,3.7)	Lema et al [24]	2019		
	2(1.58, 7.98)	Dadi et al [26]	2014		
	3.3(2,5.4)	Geleta et al [27]	2016		
	2.37(0.16,1.08)	Shibre et al [10]	2015		
	3.3(1.27,8.3)	Tegenu et al [28]	2018		
	4.2(1.07,16.6)	Abuka et al [29]	2017		
	1.64(0.36,0.93)	Workineh et al [30]	2017		
	6.10(2.5,14.93)	Markos et al [31]	2019		
	8.33(2.6.3,10.50)	Gedefaw et al [32]	2015		
Child history of Acute Respiratory Tract infection (ARTI)	1.56(0.79,3.06)	Negash AA et al [22]	2019	2.62 (1.68, 3.56)	11.7% (0.337)
	1.36(0.26,7.21)	Abaye et al [23]	2019		
	4.26(1.56,11.59)	Lema et al [24]	2019		
	3.04(1.2,7.77)	Dadi et al [26]	2014		
	5.2(3.1,8.9)	Geleta et al [27]	2016		
	4.03(2, 8)	Tegenu et al [28]	2018		
	2.75(1.3,5.81)	Lenda et al [35]	2018		
	2.71(1.12,6.52)	Onyango et al [39]	2012		
	17.13(5.01,60.26)	Muthumbi et al [40]	2017		

### Use of wood as fuel source

Eight studies found significant association between use of wood as fuel source and pneumonia among under five children. Of these the highest risk factor, AOR = 7.41 (95% CI: 2.75, 19.95), Fekadu et al [25] and lowest risk factor AOR = 1.15(0.47,1.88),Negash et al [22] compared to those who use non wood items as a source of fuel (Table 3). Regarding heterogeneity test, Galbraith plot showed homogeneity and combining the result of eight studies, the forest plot showed the overall estimate of AOR of using wood as fuel source was 1.53(95%C I: 1.30, 1.77;I^2^ = 0.0%;*P* = 0.465). I-Squared (I^2^) and *P*-value also showed homogeneity (Supplementary Fig. 6). Regarding publication bias, a funnel plot showed a symmetrical distribution. During the Egger’s regression test, the *p*-value was 0.176, which indicated the absence of publication bias (Supplementary Fig. 7).

We employed a leave-one-out sensitivity analysis to identify the potential source of heterogeneity in the analysis of the pooled estimate of using wood as fuel source as a risk factor of pneumonia in Eastern Africa. The results of this sensitivity analysis showed that our findings were not dependent on a single study. Our pooled estimate of using wood as fuel source varied between 1.409(95% CI, 1.122–1.696) and 1.664 (95% CI, 1.321–2.008) after deletion of a single study (Supplementary Fig. 8).

### Cooking food in living room

Six studies found significant association between cooking food at living room and pneumonia among under five children. Of these the highest risk factors, AOR = 3.27(1.4, 7.9) Tegenu et al [28] and lowest risk factor AOR = 1.35(0.3,0.99) Sikolia et al [43] compared to those who cook food at kitchen (Table 3). Regarding heterogeneity test for cooking food at in living room, Galbraith plot showed homogeneity and combining the result of six studies the forest plot showed the overall estimate of AOR of cooking food in living room was 1.47(95%CI: 1.16–1.79;I^2^ = 0.0%;*P* = 0.58).I-Squared (I^2^) and *P*-value also showed homogeneity (Supplementary Fig. 9). Regarding publication of bias for cooking food at home, the funnel plot analysis showed asymmetrical distribution. During the Egger’s regression test, the *p*-value was 0.026, which indicated the presence of publication bias (Supplementary Fig. 10). Trim and fill analysis was done, and 3 study were added and the total number of studies become 9. The pooled estimate of AOR of preterm becomes 1.406 (Supplementary Fig. 11). We employed a leave-one-out sensitivity analysis to identify the potential source of heterogeneity in the analysis of the pooled estimate of cooking food in living room as a risk factor of pneumonia in Eastern Africa. The results of this sensitivity analysis showed that our findings were not dependent on a single study. Our pooled estimate of cooking food in living room varied between 1.428(95%CI, 1.102–1.755) and 2.09(95%CI, 1.314–2.875) after deletion of a single study (Supplementary Fig. 12).

### Caring of the child on mothers during cooking

Seven studies found significant association between putting a child at the back during cooking and pneumonia among under five children. Of these the highest risk factors, AOR = 11.76(4.6, 30.08) Lema et al [24] and lowest risk factor AOR = 1.37(0.24,7.83) Abuka et al [29] compared to those who didn’t put their baby at their back (Table 3). Regarding heterogeneity test, Galbraith plot showed homogeneity and combining the result of seven studies the forest plot showed the overall estimate of AOR of pneumonia was 3.26(95%CI: 1.80–4.72;I^2^ = 22.5%;*P* = 0.258).I-Squared (I^2^) and *P*-value also showed homogeneity (Supplementary Fig. 13). Regarding test of publication bias a funnel plot showed a symmetrical distribution. Egger’s regression test *p*-value was 0.074, which indicated the presence of publication bias (Supplementary Fig. 14). We employed a leave-one-out sensitivity analysis to identify the potential source of heterogeneity in the analysis of the pooled estimate of putting a child at the back during cooking as a risk factor of pneumonia in Eastern Africa. The results of this sensitivity analysis showed that our findings were not dependent on a single study. Our pooled estimate of putting a child at the back during cooking varied between 2.87(95% CI, 1.329–4.426) and 3.59(95% CI, 1.828–5.355) after deletion of a single study (Supplementary Fig. 16).

### Being unvaccinated

Seven studies found significant association between being unvaccinated and pneumonia among under five children. Of these the highest risk factors, AOR = 4.62(2.64, 11) Tegenu et al [28] and lowest risk factor AOR = 1.6(0.9,2.9) Geleta et al [27] compared to those who have been vaccinated (Table 3). Regarding heterogeneity test, Galbraith plot showed homogeneity and combining the result of seven studies, the forest plot showed the overall estimate of AOR of not being vaccinated was 2.41(95%C I: 2.00–2.81;I^2^ = 51.4%;*P* = 0.055).I-Squared (I^2^) and *P*-value also showed homogeneity (Supplementary Fig. 17). Regarding publication bias, a funnel plot showed a symmetrical distribution. During the Egger’s regression test, the *p*-value was 0.177, which indicated the absence of publication bias (Supplementary Fig. 18). We employed a leave-one-out sensitivity analysis to identify the potential source of heterogeneity in the analysis of the pooled estimate of being unvaccinated as a risk factor of pneumonia in Eastern Africa. The results of this sensitivity analysis showed that our findings were not dependent on a single study. Our pooled estimate of being unvaccinated varied between 2.4(95%CI, 2.07–2.72) and 2.71(95%CI, 2.55–2.86) after deletion of a single study (Supplementary Fig. 19).

### Non-exclusive breast feeding

Eleven studies found significant association between non-exclusive breast feeding and pneumonia among under five children. Of these the highest risk factors, AOR = 8.33(2.6.3,10.50) Gedefaw et al [32] and lowest risk factor AOR = 1.51(0.88,2.58) Negash et al [22] compared to those who breast feed exclusively (Table 3). Regarding heterogeneity test, Galbraith plot showed heterogeneity and combining the result of eleven studies, the forest plot showed the overall estimate of AOR of non-exclusive breast feeding was 2.47(95%C I: 1.79, 3.16;I^2^ = 65.0%;*P* = 0.01).I-Squared (I^2^) and *P*-value also showed heterogeneity (Supplementary Fig. 20). Regarding publication bias, a funnel plot showed an asymmetrical distribution. During the Egger’s regression test, the *p*-value was 0.016, which indicated the presence of publication bias (Supplementary Fig. 21). Due to presence of publication bias trim and fill analysis was done and 5 studies were added, and the total number of studies becomes 16. The pooled estimate of AOR of non-exclusive breast feeding was found to be 2.05 (Supplementary Fig. 22). We employed a leave-one-out sensitivity analysis to identify the potential source of heterogeneity in the analysis of the pooled estimate of being non-exclusive breast feeding as a risk factor of pneumonia in Eastern Africa. The results of this sensitivity analysis showed that our findings were not dependent on a single study. Our pooled estimate of being for non-exclusive breast feeding is found to be between 1.757(95%CI, 1.49–2.01) and 1.936(95%CI, 1.70–2.17) after deletion of a single study (Supplementary Fig. 23).

### History acuter respiratory tract infection (ARTI)

History ARTI was considered when a child has history of ARTI with in the 2 weeks before being diagnosed for pneumonia. Nine studies found significant association between history ARTI and pneumonia among under five children. Of these the highest risk factors, AOR = 17.13(5.01,60.26) Muthumbi et al [40] and lowest risk factor AOR = 1.36(0.26,7.21) Abaye et al [23] compared to those who use non wood item as a source of fuel (Table 3). Regarding heterogeneity test, Galbraith plot showed homogeneity and combining the result of nine studies, the forest plot showed the overall estimate of AOR of history ARTI was considered was 2.62(95%C I: 1.68, 3.56;I^2^ = 11.7%;*P* = 0.337).I-Squared (I^2^) and *P*-value also showed homogeneity (Supplementary Fig. 24). Regarding publication bias, a funnel plot showed an asymmetrical distribution. During the Egger’s regression test, the *p*-value was 0.024, which indicated the presence of publication bias (Supplementary Fig. 25). Due to presence of publication bias trim and fill analysis was done and 5 studies were added, and the total number of studies becomes 14. The pooled estimate of AOR of history of ARTI was found to be 1.958(Supplementary Fig. 26).

We employed a leave-one-out sensitivity analysis to identify the potential source of heterogeneity in the analysis of the pooled estimate of being history of ARTI as a risk factor of pneumonia in Eastern Africa. The results of this sensitivity analysis showed that our findings were not dependent on a single study. Our pooled estimate of having history of ARTI ranges between 2.195(95%CI, 1.36–3.02) and 3.28(95%CI, 2.153–4.417) after deletion of a single study (Supplementary Fig. 27).

## Discussion

This systematic review and meta-analysis was conducted to assess the magnitude of pneumonia and its associated factors among under-five children in East Africa. Thirty-four studies were included for the final analysis. Twenty-two studies had reported the prevalence of pneumonia and the pooled prevalence of pneumonia in under-five children was found to be 34% with 95% CI of (23.8–44.21%). This result was higher than a study conducted in Dibrugarh, India which had reported the prevalence of pneumonia in under-five children to be 16.34% [9]. This might be due to socioeconomic and seasonal discrepancies as countries in East Africa are less developed than India. A study conducted in Nigeria had revealed the prevalence of pneumonia in under-five children to be 31.6% which was consistence with the findings of this systematic review [53]. This consistency might be due to similarities in socio-economic status as Nigeria is an African country probably having comparable socio-economic status with east African countries. In addition the discrepancy might be due to difference in case definition of pneumonia.

This finding is higher than other studies done in Austria (4.1%) [54], in Mali (6.7%) [55], and in Bangladesh (21.3%) [56]. This variation might be due to socio-economic and socio-demographic vitiations, the variation in the study setting, seasonal variation, unreachability and provision of Vitamin A supplementation and immunization, lack of confirmatory laboratories and imaging investigations.

This systematic review and meta-analysis had also revealed using woods as a source of fuel, cooking foods living rooms, holding children on back while cooking foods, being unvaccinated, history of being not on exclusive breast feeding, history of upper respiratory tract infection and parental smoking as a significant risk factors for increased prevalence of pneumonia among under-five children in East Africa.

Higher odds of pneumonia were observed in under-five children whose family uses wood as a source of fuel. This result was in line with studies conducted in India [57], and Sri Lanka [58]; and with systematic reviews conducted in Low and Middle income countries [59], and Africa, China and Latin America [60]. It was also consistent with a global review conducted by Jackson et al. [61]. The association between using wood as a source of fuel and pneumonia in under-five children might be due to the fact that using woods as a source of fuel results in release of wood smokes containing major air pollutants like carbon monoxide and particulate matters which causes indoor air pollution [62]. Indoor air pollution and inhaling wood smoke in turn impairs the function of pulmonary alveolar macrophages and epithelial cells which will increase the likelihood of pulmonary infections including pneumonia [62, 63].

According to this systematic review and meta-analysis, cooking foods in living rooms was found to be significantly associated with occurrence of pneumonia in under-five children as higher odds of pneumonia was exhibited among children living in families who cooks food at living rooms than children living in families who cooks food in kitchen. Holding children on back while cooking foods was another factor found to be significantly associated with pneumonia. This association might be due to the reason that cooking foods in living rooms will cause indoor air pollution and holding a child on back while cooking foods can increase the probability of inhaling smokes and food vapors (steams) which in turn will increase the risk of acquiring pneumonia by altering the structure and function of the respiratory tract [58, 63].

In this systematic review children with history of Acute Respiratory Tract Infections (ARTIs) were found to be at increased risk to acquire pneumonia; as the odds of pneumonia among children who had history of ARTIs was higher than children without history of ARTIs. The reason behind this association might be due to the fact that ARTIs will alter the structure and function of the respiratory tract and can cause Lower Respiratory Infections (LRTIs) including pneumonia in two ways— by increasing invasion of the Lower respiratory tract (LRT) with other microorganisms which cause secondary infections or by progressive invasion of LRT with the same microorganism causing the ARTIs (Primary infections) [64].

The risk of acquiring pneumonia in unvaccinated children was found to be higher than vaccinated children. This result was similar with studies conducted in Brazil [65], Bellary [7], and India [66]. A systematic review conducted by Jackson et al. [61] was also in line with this result. Similarly, children who were not on exclusive breast feeding were at higher risk to develop pneumonia than children who were on exclusive breast feeding for the first 6 months of age. This result was consistent with different studies conducted across the world [7, 61, 67, 68]. The reason behind this association might be due to low or weak immunity. Because exclusive breast feeding and vaccination are strategies used to increase the immunity of children and prevent childhood infections. So, children who were not on Exclusive breast feeding and/ or unvaccinated will have weak immunity and increased probability of acquiring infections including pneumonia [69].

### Strength and limitations

This study has several strengths: First, we used a pre-specified protocol for search strategy and data abstraction and used internationally accepted tools for a critical appraisal system for quality assessment of individual studies. Second, we employed subgroup and sensitivity analysis based on study country, study design, and publication year to identify the small study effect and the risk of heterogeneity. Nevertheless, this review had some limitations: There may be publication bias because not all grey literature was included and language biases since all included studies are published in English.

## Conclusion and recommendation

The prevalence of pneumonia among under-five children in Eastern Africa remains high. Use of wood as fuel source, cooking food in living room, caring of a child on mother during cooking, being unvaccinated, on-exclusive breast feeding,child history of ARTI, and parental smoking were independent potential predictors of under-five pneumonia in Eastern Africa. Hence, appropriate intervention on potential determinates such as health education on exclusive breastfeeding, place of food cooking, increase vaccination coverage and early control of respiratory tract infection was recommended to prevent those risk factors.

## Supplementary information

**Additional file 1.** PRISMA 2009 Checklist

**Additional file 2 Supplementary Figure 1**. Forest plot showing subgroup analysis (by country) of pooled prevalence of pneumonia among under-five children in Ethiopia from2002 up to 2019. **Supplementary Figure 2**. Forest plot showing subgroup analysis (by study design) of pooled prevalence of pneumonia among under-five children in Ethiopia from2002 up to 2019. **Supplementary Figure 3**. Forest plot showing subgroup analysis (by country) of pooled prevalence of pneumonia among under-five children in Ethiopia from2002 up to 2019. **Supplementary Figure 4**. sensitivity of pooled prevalence of pneumonia among under-five children in Ethiopia from2002 up to 2019. **Supplementary Figure 5**. publication bias of pooled prevalence of pneumonia among under-five children in Ethiopia from2002 up to 2019. **Supplementary Figure 6**. Forest plot showing of pooled estimate of AOR for using wood as fuel source as a predictor of pneumonia among under-five children in Ethiopia from2002 up to 2019. **Supplementary Figure 7**. publication bias of pooled estimate of AOR for using wood as fuel source as a predictor of pneumonia among under-five children in Ethiopia from2002 up to 2019. **Supplementary Figure 8**. sensitivity analysis of pooled estimate of AOR for using wood as fuel source as a predictor of pneumonia among under-five children in Ethiopia from2002 up to 2019. **Supplementary Figure 9**: Forest plot showing the pooled estimate of AOR for cooking food at home as a predictor of pneumonia among under-five children in Ethiopia from2002 up to 2019.**Supplementary Figure 10**. publication bias for pooled estimate of AOR for cooking food at home as a predictor of pneumonia among under-five children in Ethiopia from2002 up to 2019. **Supplementary Figure 11**. Trim and fill analysis for pooled estimate of AOR for cooking food at home as a predictor of pneumonia among under-five children in Ethiopia from2002 up to 2019. **Supplementary Figure 12**. Sensitivity analysis for pooled estimate of AOR for cooking food at home as a predictor of pneumonia among under-five children in Ethiopia from2002 up to 2019. **Supplementary Figure 13.** Forest plot showing estimate of AOR for caring of the child on mothers during cooking as a predictor of pneumonia among under-five children in Ethiopia from2002 up to 2019. **Supplementary Figure 14**. publication bias for estimate of AOR for caring of the child on mothers during cooking as a predictor of pneumonia among under-five children in Ethiopia from2002 up to 2019. **Supplementary Figure 15**. trim and fill analysis for estimate of AOR for caring of the child on mothers during cooking as a predictor of pneumonia among under-five children in Ethiopia from2002 up to 2019. **Supplementary Figure 16**. sensitivity analysis for estimate of AOR for caring of the child on mothers during cooking as a predictor of pneumonia among under-five children in Ethiopia from2002 up to 2019. **Supplementary Figure 17**. Forest plot showing the pooled estimate of AOR for being unvaccinated as a predictor of pneumonia among under-five children in Ethiopia from2002 up to 2019. **Supplementary Figure 18**. publication bias for pooled estimate of AOR for being unvaccinated as a predictor of pneumonia among under-five children in Ethiopia from2002 up to 2019. **Supplementary Figure 19**. sensitivity analysis for pooled estimate of AOR for being unvaccinated as a predictor of pneumonia among under-five children in Ethiopia from2002 up to 2019. **Supplementary Figure 20**. Forest plot showing the pooled estimate of AOR for non-exclusive breast feeding as a predictor of pneumonia among under-five children in Ethiopia from 2002 up to 2019. **Supplementary Figure 21**. publication bias for the pooled estimate of AOR for non-exclusive breast feeding as a predictor of pneumonia among under-five children in Ethiopia from 2002 up to 2019. **Supplementary Figure 22**. Trim and fill analysis for the pooled estimate of AOR for non-exclusive breast feeding as a predictor of pneumonia among under-five children in Ethiopia from 2002 up to 2019. **Supplementary Figure 23**. Sensitivity analysis for the pooled estimate of AOR for non-exclusive breast feeding as a predictor of pneumonia among under-five children in Ethiopia from 2002 up to 2019. **Supplementary Figure 24**. Forest plot showing the pooled estimate of AOR for history of ARTI as a predictor of pneumonia among under-five children in Ethiopia from 2002 up to 2019. **Supplementary Figure 25**. Publication bias for the pooled estimate of AOR for history of ARTI as a predictor of pneumonia among under-five children in Ethiopia from 2002 up to 2019. **Supplementary Figure 26**. Trim and fill analysis for the pooled estimate of AOR for history of ARTI as a predictor of pneumonia among under-five children in Ethiopia from 2002 up to 2019. **Supplementary Figure 27**. sensitivity analysis for the pooled estimate of AOR for history of ARTI as a predictor of pneumonia among under-five children in Ethiopia from 2002 up to 2019

**Additional file 3 Table S1**. Search strategy used for one of the databases

**Additional file 4 Table S2**. Quality appraisal result of included studies in East Africa, from 2002 to 2019. Using Joanna Briggs Institute (JBI) quality appraisal checklist

**Additional file 5 Table S3**. Adjusted confounders and main findings extracted from included studies in East Africa

## Data Availability

The datasets analyzed during the current study are available from the corresponding author upon reasonable request.

## Abbreviations

CI: Confidence Interval
OR: Odds Ratio
U5M: Under Five Mortalities
WHO: World Health Organization
DHS: Demographic and Health Surveys
EDHS: Ethiopian Demographic and Health Survey
AOR: Adjusted odds ratio
ARTI: Acute Respiratory Tract Infections
