# Supplementary material for: Prevalence of pneumonia and its associated factors among under-five children in East Africa: a systematic review and meta-analysis
Source: BMC Pediatr. 2020 May 27;20:254. doi: 10.1186/s12887-020-02083-z (PMC7251746; doi:10.1186/s12887-020-02083-z)
Supplement: Supplementary file 1 — Additional file 1. PRISMA 2009 Checklist [file 12887_2020_2083_MOESM1_ESM.doc]

| **Section/topic** | **#** | **Checklist item** | **Reported on page #** |
| --- | --- | --- | --- |
| **TITLE** | | |  |
| Title | 1 | **Prevalence of pneumonia and its associated factors among under five children in East Africa. Meta-analysis and systemic review** | 1 |
| **ABSTRACT** | | |  |
| Structured summary | 2 | Background: Pneumonia is defined as an acute inflammation of the Lungs’ parenchymal structure. It is a major public health problem and the leading cause of morbidity and mortality in under-five children especially in developing countries. In 2015, it was estimated that about 102 million cases of pneumonia occurred in under-five children, of which 0.7 million were end up with death. Different primary studies in Eastern Africa showed the burden of pneumonia. However, inconsistency among those studies was seen and no review has been conducted to report the amalgamated magnitude and associated factors. Therefore, this review aimed to estimate the national prevalence and associated factors of neonatal sepsis in Eastern Africa.  Objective: The aim of this systematic review and meta-analysis was to assess the magnitude of pneumonia and its associated factors among under-five children in East Africa.  Methods: Using PRISMA guideline, we systematically reviewed and meta-analyzed studies that examined the prevalence and associated factors of pneumonia from PubMed, Cochrane library, and Google Scholar. Heterogeneity across the studies was evaluated using the Q and the I2 test. A weighted inverse variance random-effects model was applied to estimate the national prevalence and the effect size of associated factors. The subgroup analysis was conducted by country, study design, and year of publication. A funnel plot and Egger’s regression test were used to see publication bias. Sensitivity analysis was also done to identify the impact of studies.  Result: A total of 34 studies with 87, 984 participants were used for analysis. The pooled prevalence of hypothermia in East Africa was 34% (95%CI; 23.80–44.21). Use of wood as fuel source (AOR= 1.53; 95%CI:1.30-1.77; I2= 0.0% ;P=0.465), cook food in living room (AOR= 1.47;95%CI:1.16-1.79; I2= 0.0% ;P=0.58), caring of a child on mother during cooking (AOR= 3.26; 95%CI:1.80-4.72; I2= 22.5% ;P=0.26), Being unvaccinated (AOR= 2.41; 95%CI:2.00-2.81; I2= 51.4% ;P=0.055), Child history of ARTI(AOR= 2.62; 95%CI:1.68-3.56; I2= 11.7% ;P=0.337) were identified factors of pneumonia.  Conclusions: The prevalence of pneumonia in Eastern Africa remains high. This review will help policy-makers and program officers to design pneumonia preventive interventions.  Keywords: Pneumonia, Eastern Africa ,Under five | 2 |
| **INTRODUCTION** | | |  |
| Rationale | 3 | In East African countries different researchers had tried to investigate the magnitude of pneumonia in under-five children and have reported a prevalence ranges from 5.5% (10) up to 89.8% (11). They had also identified risk factors for pneumonia among under-five children. But, reported finding lack consistency and as per the investigators knowledge there is no a systematic review and meta-analysis conducted to address these inconsistent findings reported from East African countries. Moreover, assessing the magnitude of pneumonia and identifying its associated factors for risk based diagnosis of pneumonia contribute in better interventions and helps to reduce the higher burden of pneumonia in under-five children. Hence, this systematic review and meta-analysis was conducted to assess the magnitude of pneumonia and its associated factors among under-five children in East Africa. | 4 |
| Objectives | 4 | To assess the pooled prevalence of pneumonia and its associated factors in East Africa | 4 |
| **METHODS** | | |  |
| Protocol and registration | 5 | Prospero database:(PROSPERO 2019:CRD42019136707) Available from https://www.crd.york.ac.uk/PROSPERO/#myprosperoID = CRD42019136707 | 4 |
| Eligibility criteria | 6 | Retrieved studies were exported to reference manager software, Endnote version 8 to remove duplicate studies. Two investigators (BB and AM) independently screened the selected studies using their titles and abstracts before retrieval of full-text papers. We used pre-specified inclusion criteria to further screen the full-text articles. Disagreements were discussed during a consensus meeting with other reviewers (MW and MB) for the final selection of studies to be included in the systematic review and meta-analysis.  Inclusion and exclusion criteria  All observational studies (cross-sectional, case-control, and cohort studies) were included. Those studies had reported the prevalence and/or at least one associated factors of pneumonia among under-five children and published in English language from 2002 up to 2019 in Eastern Africa were considered. A consideration was extended to unpublished work among children under five were also considered. Citations without abstract and/or full-text, anonymous reports, editorials, and qualitative studies were excluded from the analysis. Furthermore, research which did not report our results of interest was excluded. | 5 |
| Information sources | 7 | PubMed, Cochrane library, and Google Scholar were accessed. Articles with incomplete reported data were handled through contacting corresponding authors | 5 |
| Search | 8 | We identified studies providing data on the prevalence of and potential risk factors of pneumonia among under-five children, with the search focused on Eastern Africa from PubMed, Cochrane library, and Google Scholar. The search included MeSH terms and keywords, combinations, and snowball searching in references list of papers found through the data base search to retrieve additional articles. Articles with incomplete reported data were handled through contacting corresponding authors. Unpublished studies were retrieved from the official websites of international and local organizations and universities. The search was performed by keywords, medical subject headings (MeSH) terms. We used the search terms independently and/or in combination using “OR” or “AND”. The core search terms and phrases were “under five”, “children”, “child”, “infant”, and “pneumonia”, “respiratory infection”, causes, risk factors, determinants, associated factors, predictors and Eastern Africa. The search strategies were developed using different Boolean operators. Remarkably, to fit advanced PubMed database, the following search strategy was applied: (prevalence OR magnitude OR epidemiology) AND (causes OR determinants OR associated factors OR predictors OR risk factors) AND (children [MeSH Terms] OR under five OR child OR childhood) AND (pneumonia [MeSH Terms] OR respiratory tract infection) AND Eastern Africa. We also screened at the reference lists of the remaining papers to identify additional relevant studies to this review | 4 and 5 |
| Study selection | 9 | Study selection / Eligibility criteria  Retrieved studies were exported to reference manager software, Endnote version 8 to remove duplicate studies. Two investigators (BB and AM) independently screened the selected studies using their titles and abstracts before retrieval of full-text papers. We used pre-specified inclusion criteria to further screen the full-text articles. Disagreements were discussed during a consensus meeting with other reviewers (MW and MB) for the final selection of studies to be included in the systematic review and meta-analysis.  Inclusion and exclusion criteria  All observational studies (cross-sectional, case-control, and cohort studies) were included. Those studies had reported the prevalence and/or at least one associated factors of pneumonia among under-five children and published in English language from 2002 up to 2019 in Eastern Africa were considered. A consideration was extended to unpublished work among children under five were also considered. Citations without abstract and/or full-text, anonymous reports, editorials, and qualitative studies were excluded from the analysis. Furthermore, research which did not report our results of interest was excluded.    Quality assessment  Duplicate articles were removed using Endnote (version X8) after combining the Database search results. The Joanna Briggs Institute (JBI) quality appraisal checklist was used (12, 13).Four independent authors appraised the quality of the studies. The appraisal was repeated by exchanging with each other. Thus, one paper was appraised by two Authors. Any disagreement between the reviewers was solved by taking the mean score of the two reviewers. Studies were considered as low risk or good quality when it scored 5 and above for all designs (cross sectional, case control, and cohort) and were included(12, 13) whereas the score was 4 and below the studies considered as high risk or poor quality and was not included.. | 5 |
| Data collection process | 10 | **Data extraction**  The authors developed data extraction form on the excel sheet which includes author name, year of publication, study country, study design, sample size, prevalence of pneumonia, and categories of factors reported. The data extraction sheet was piloted using 4 papers randomly. The extraction form was adjusted after piloted the template. Two of the authors extracted the data using the extraction form in collaboration. The third and fourth authors check the correctness of the data independently. Any disagreements between reviewers were resolved through discussions with a third reviewer and fourth reviewer if required. The mistyping of data was resolved through crosschecking with the included papers. If we got incomplete data, we excluded the study after two attempts were made to contact the corresponding author by email. | 6 |
| Data items | 11 | Pneumonia was considered when under five children with cough and/or difficulty of breathing and have fast breathing and/or chest endowing | 6 |
| Risk of bias in individual studies | 12 | The prevalence of pneumonia were ranged from 5.5% (10) up to 89.8% (11). The random-effects model analysis from those studies revealed that, the pooled prevalence of pneumonia in East Africa was found to be 34% (95%CI; 23.80–44.21; I^2^=99.4%; p<0.001)the studies (I^2^=99.5%; p<0.001). We analyzed by random-effects model analysis and we did subgroup analysis. | 7 |
| Summary measures | 13 | We extracted and assessed the prevalence of pneumonia and odds ratio | 7 |
| Synthesis of results | 14 | **Statistical analysis**  After the data was extracted using Microsoft Excel format we imported the data to STATA version 14.0 statistical software for further analysis. Using the binomial distribution formula, Standard error was calculated for each study. We pooled the overall magnitude estimates of pneumonia by a random effect meta-analysis (14).The pooled prevalence of pneumonia with 95% CI was presented using forest plots and Odds ratio (OR) with 95% CI was also presented in forest plot to show the associated factors of pneumonia. We examined the heterogeneity between the studies using Cochrane's Q statistics (Chi-square), invers variance (I2) and p-values (15).  In this study, the I^2^ statistic value of zero indicates true homogeneity, whereas the value 25, 50, and 75% represented low, moderate and high heterogeneity respectively (16, 17). For the data identified as heterogeneous, we conducted our analysis by random-effects model analysis. In addition subgroup analysis was done by the study country, design, and year of publication. When statistical pooling is not possible, non-pooled data was presented in table form. Sensitivity analysis was employed to see the effect of a single study on the overall estimation. Publication bias was checked by funnel plot and more objectively through Egger’s regression test (18). | 7 |

Page 1 of 2

| **Section/topic** | **#** | **Checklist item** | **Reported on page #** |
| --- | --- | --- | --- |
| Risk of bias across studies | 15 | The prevalence of pneumonia were ranged from 5.5% (10) up to 89.8% (11). The random-effects model analysis from those studies revealed that, the pooled prevalence of pneumonia in East Africa was found to be 34% (95%CI; 23.80–44.21; I^2^=99.4%; p<0.001)the studies (I^2^=99.5%; p<0.001). We analyzed by random-effects model analysis and we did subgroup analysis.  . | 7 |
| Additional analyses | 16 | **Statistical analysis**  After the data was extracted using Microsoft Excel format we imported the data to STATA version 14.0 statistical software for further analysis. Using the binomial distribution formula, Standard error was calculated for each study. We pooled the overall magnitude estimates of pneumonia by a random effect meta-analysis (14).The pooled prevalence of pneumonia with 95% CI was presented using forest plots and Odds ratio (OR) with 95% CI was also presented in forest plot to show the associated factors of pneumonia. We examined the heterogeneity between the studies using Cochrane's Q statistics (Chi-square), invers variance (I2) and p-values (15).  In this study, the I^2^ statistic value of zero indicates true homogeneity, whereas the value 25, 50, and 75% represented low, moderate and high heterogeneity respectively (16, 17). For the data identified as heterogeneous, we conducted our analysis by random-effects model analysis. In addition subgroup analysis was done by the study country, design, and year of publication. When statistical pooling is not possible, non-pooled data was presented in table form. Sensitivity analysis was employed to see the effect of a single study on the overall estimation. Publication bias was checked by funnel plot and more objectively through Egger’s regression test (18). | 7 |
| **RESULTS** | | |  |
| Study selection | 17 | A total of 6879 studies were identified using electronic searches (through Databases searching (n = 6867)) and other sources (n =12)) that were conducted from 2014 up to 2019. After duplication removed, a total of 3150 articles remained (3729 duplicated). Finally, 200 studies were screened for full-text review and, 34 articles with (n=87,984 patients) were selected for the prevalence and/ or associated factors analysis. | 7 |
| Study characteristics | 18 | Table 1 summarizes the characteristics of the 34 included studies in the systematic review and meta-analysis (10, 11, 19-49). 16 studies were found in Ethiopia(10, 20-34), 8 in Kenya (11, 35-40), 2 in Uganda(48, 49),1 Eritrea(19), 1 in Somali(41),4 Sudan(42-45),2 Tanzania(46, 47).  23 studies were cross-sectional, while the others used either case-control (n=9) or cohort (n=2) study design. Most of the studies 23/34(70.5%) were published between 2015 and 2019. The studies included participants, ranging from 40 (45) to 73778(41) (Table1). | 8 |
| Risk of bias within studies | 19 | We have also checked publication bias and a funnel plot showed symmetrical distribution. Egger’s regression test p-value was 0.63, which indicated the absence of publication bias | 13 |
| Results of individual studies | 20 | The prevalence of pneumonia were ranged from 5.5% (10) up to 89.8% (11). The random-effects model analysis from those studies revealed that, the pooled prevalence of pneumonia in East Africa was found to be 34% (95%CI; 23.80–44.21; I^2^=99.4%; p<0.001) | 10 |
| Synthesis of results | 21 | We found significant heterogeneity among the studies (I^2^=99.4%; p<0.001). We analyzed by random-effects model analysis and we did subgroup analysis. Publication bias was checked by funnel plot and more objectively through Egger’s regression test . | 10 |
| Risk of bias across studies | 22 | We employed a leave-one-out sensitivity analysis to identify the potential source of heterogeneity in the analysis of the prevalence of pneumonia in Eastern Africa. The results of this sensitivity analysis showed that our findings were not dependent on a single study. Our pooled estimated prevalence of pneumonia varied between 31.38(22.93–39.83)(11) and 35.3(25.13–45.49)(10) after deletion of a single study | 13 |
| Additional analysis | 23 | The subgroup analysis was done through stratified by country, study design, and year of publication. Based on this, the prevalence of pneumonia among under five children was found to be 29 in Eritrea, 22.62 in Ethiopia, 64.3 in Kenya, 29.71 in Sudan, 22 in Tanzania, and 32.72 in Uganda (Supplementary Fig 1 and Table 2). Based on the study design, the prevalence of pneumonia was found to be 32.33 in cross-sectional studies, 55.68% in cohort studies and 22.6 in case control studies (Supplementary Fig 2 and Table 2). Based on the year of publication, the prevalence of pneumonia was found to be 33.4 from 2000-2015, while it was 34.29 from studies conducted from 2016-2019 | 19-21 |
| **DISCUSSION** | | |  |
| Summary of evidence | 24 | This systematic review and meta-analysis was conducted to assess the magnitude of pneumonia and its associated factors among under-five children in East Africa. Thirty-four studies were included for the final analysis. Twenty-two studies had reported the prevalence of pneumonia and the pooled prevalence of pneumonia in under-five children was found to be 34% with 95% CI of (23.8- 44.21%). This result was higher than a study conducted in Dibrugarh, India which had reported the prevalence of pneumonia in under-five children to be 16.34% (9). This might be due to socioeconomic discrepancies as countries in East Africa are less developed than India. A study conducted in Nigeria had revealed the prevalence of pneumonia in under-five children to be 31.6% which was consistence with the findings of this systematic review (51). This consistency might be due to similarities in socio-economic status as Nigeria is an African country probably having comparable socio-economic status with east African countries.  This systematic review and meta-analysis had also revealed using woods as a source of fuel, cooking foods living rooms, holding children on back while cooking foods, being unvaccinated, history of being not on exclusive breast feeding, history of upper respiratory tract infection and parental smoking as a significant risk factors for increased prevalence of pneumonia among under-five children in East Africa.  Higher odds of pneumonia were observed in under-five children whose family uses wood as a source of fuel. This result was in line with studies conducted in India (52), and Sri Lanka (53); and with systematic reviews conducted in Low and Middle income countries (54), and Africa, China and Latin America (55). It was also consistent with a global review conducted by Jackson et al. (56). The association between using wood as a source of fuel and pneumonia in under-five children might be due to the fact that using woods as a source of fuel results in release of wood smokes containing major air pollutants like carbon monoxide and particulate matters which causes indoor air pollution (57). Indoor air pollution and inhaling wood smoke in turn impairs the function of pulmonary alveolar macrophages and epithelial cells which will increase the likelihood of pulmonary infections including pneumonia (57, 58).  According to this systematic review and meta-analysis, cooking foods in living rooms was found to be significantly associated with occurrence of pneumonia in under-five children as higher odds of pneumonia was exhibited among children living in families who cooks food at living rooms than children living in families who cooks food in kitchen. Holding children on back while cooking foods was another factor found to be significantly associated with pneumonia. This association might be due to the reason that cooking foods in living rooms will cause indoor air pollution and holding a child on back while cooking foods can increase the probability of inhaling smokes and food vapors (steams) which in turn will increase the risk of acquiring pneumonia by altering the structure and function of the respiratory tract (53, 58).  In this systematic review children with history of Upper Respiratory Tract Infections (URTIs) were found to be at increased risk to acquire pneumonia; as the odds of pneumonia among children who had history of URTIs was higher than children without history of URTIs. The reason behind this association might be due to the fact that URTIs will alter the structure and function of the respiratory tract and can cause Lower Respiratory Infections (LRTIs) including pneumonia in two ways— by increasing invasion of the Lower respiratory tract (LRT) with other microorganisms which cause secondary infections or by progressive invasion of LRT with the same microorganism causing the URTIs (Primary infections) (59).  The risk of acquiring pneumonia in unvaccinated children was found to be higher than vaccinated children. This result was similar with studies conducted in Brazil (60), Bellary (7), and India (61). A systematic review conducted by Jackson et al. (56) was also in line with this result. Similarly, children who were not on exclusive breast feeding were at higher risk to develop pneumonia than children who were on exclusive breast feeding for the first 6 months of age. This result was consistent with different studies conducted across the world (7, 56, 62, 63). The reason behind this association might be due to low or weak immunity. Because exclusive breast feeding and vaccination are strategies used to increase the immunity of children and prevent childhood infections. So, children who were not on Exclusive breast feeding and/ or unvaccinated will have weak immunity and increased probability of acquiring infections including pneumonia (64). | 30 |
| Limitations | 25 | This study has several strengths: First, we used a pre-specified protocol for search strategy and data abstraction and used internationally accepted tools for a critical appraisal system for quality assessment of individual studies.Second, we employed subgroup and sensitivity analysis based on study country, study design, and publication year to identify the small study effect and the risk of heterogeneity. Nevertheless, this review had some limitations: There may be publication bias because not all grey literature was included and language biases since all included studies are published in English. | 21 |
| Conclusions | 26 | **Conclusion and Recommendation**  The prevalence of pneumonia among under-five children in Eastern Africa remains high. Use of wood as fuel source, cooking food in living room, caring of a child on mother during cooking, being unvaccinated, on-exclusive breast feeding ,child history of ARTI, and parental smoking were independent potential predictors of under-five pneumonia in Eastern Africa. Hence, appropriate intervention on potential determinates such as health education on exclusive breastfeeding, place of food cooking, increase vaccination coverage and early control of respiratory tract infection was recommended to prevent those risk factors | 21 |
| **FUNDING** | | |  |
| Funding | 27 | N/A |  |

*From:*  Moher D, Liberati A, Tetzlaff J, Altman DG, The PRISMA Group (2009). Preferred Reporting Items for Systematic Reviews and Meta-Analyses: The PRISMA Statement. PLoS Med 6(7): e1000097. doi:10.1371/journal.pmed1000097

For more information, visit: **www.prisma-statement.org**.

Page 2 of 2
