# Supplementary material for: Prevalence of pneumonia and its associated factors among under-five children in East Africa: a systematic review and meta-analysis
Source: BMC Pediatr. 2020 May 27;20:254. doi: 10.1186/s12887-020-02083-z (PMC7251746; doi:10.1186/s12887-020-02083-z)
Supplement: Supplementary file 2 — Additional file 2 Supplementary Figure 1. Forest plot showing subgroup analysis (by country) of pooled prevalence of pneumonia among under-five children in Ethiopia from2002 up to 2019. Supplementary Figure 2. Forest plot showing subgroup analysis (by study design) of pooled prevalence of pneumonia among under-five children in Ethiopia from2002 up to 2019. Supplementary Figure 3. Forest plot showing subgroup analysis (by country) of pooled prevalence of pneumonia among under-five children in Ethiopia from2002 up to 2019. Supplementary Figure 4. sensitivity of pooled prevalence of pneumonia among under-five children in Ethiopia from2002 up to 2019. Supplementary Figure 5. publication bias of pooled prevalence of pneumonia among under-five children in Ethiopia from2002 up to 2019. Supplementary Figure 6. Forest plot showing of pooled estimate of AOR for using wood as fuel source as a predictor of pneumonia among under-five children in Ethiopia from2002 up to 2019. Supplementary Figure 7. publication bias of pooled estimate of AOR for using wood as fuel source as a predictor of pneumonia among under-five children in Ethiopia from2002 up to 2019. Supplementary Figure 8. sensitivity analysis of pooled estimate of AOR for using wood as fuel source as a predictor of pneumonia among under-five children in Ethiopia from2002 up to 2019. Supplementary Figure 9: Forest plot showing the pooled estimate of AOR for cooking food at home as a predictor of pneumonia among under-five children in Ethiopia from2002 up to 2019.Supplementary Figure 10. publication bias for pooled estimate of AOR for cooking food at home as a predictor of pneumonia among under-five children in Ethiopia from2002 up to 2019. Supplementary Figure 11. Trim and fill analysis for pooled estimate of AOR for cooking food at home as a predictor of pneumonia among under-five children in Ethiopia from2002 up to 2019. Supplementary Figure 12. Sensitivity analysis for pooled estimate of AOR for cooking food at home as [file 12887_2020_2083_MOESM2_ESM.docx]

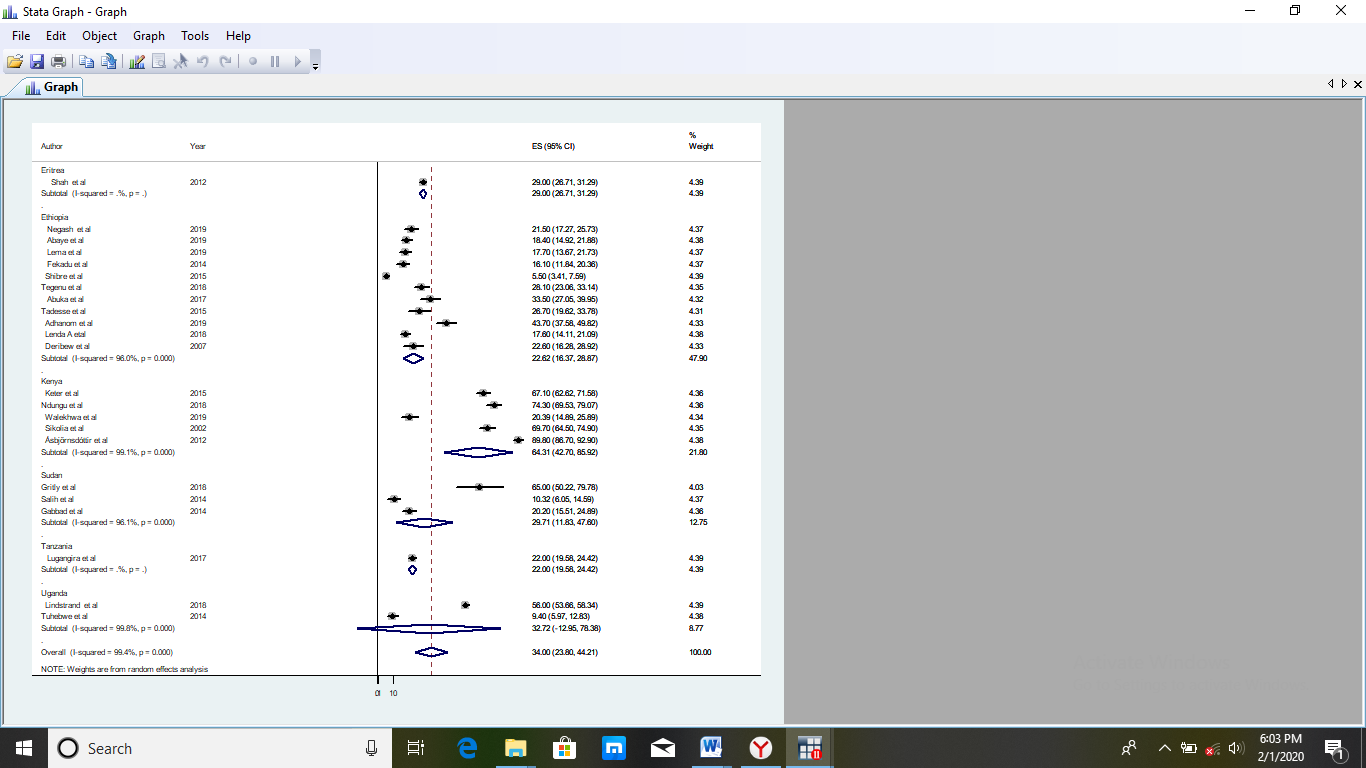


Supplementary Figure 1: Forest plot showing subgroup analysis (by country) of pooled prevalence of pneumonia among under-five children in Ethiopia from2002 up to 2019.


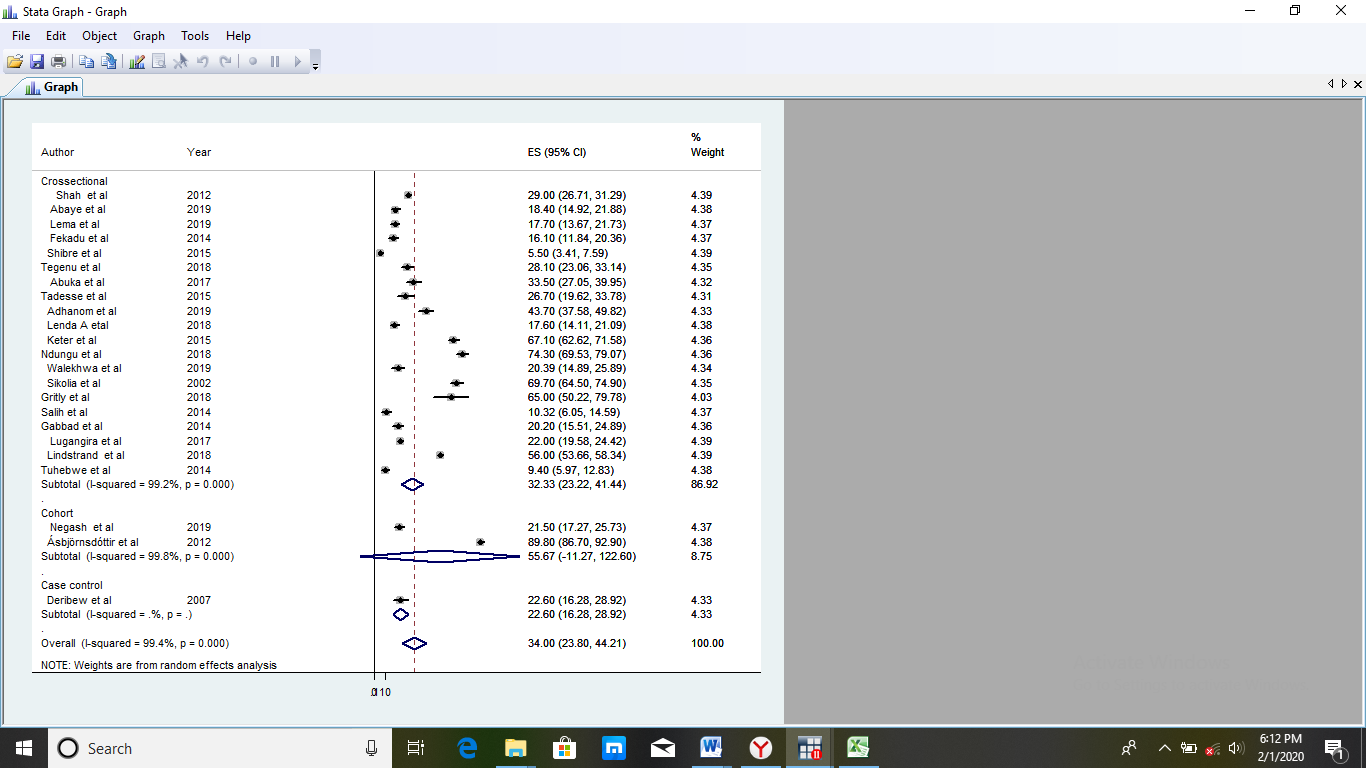

Supplementary Figure 2: Forest plot showing subgroup analysis (by study design) of pooled prevalence of pneumonia among under-five children in Ethiopia from2002 up to 2019.


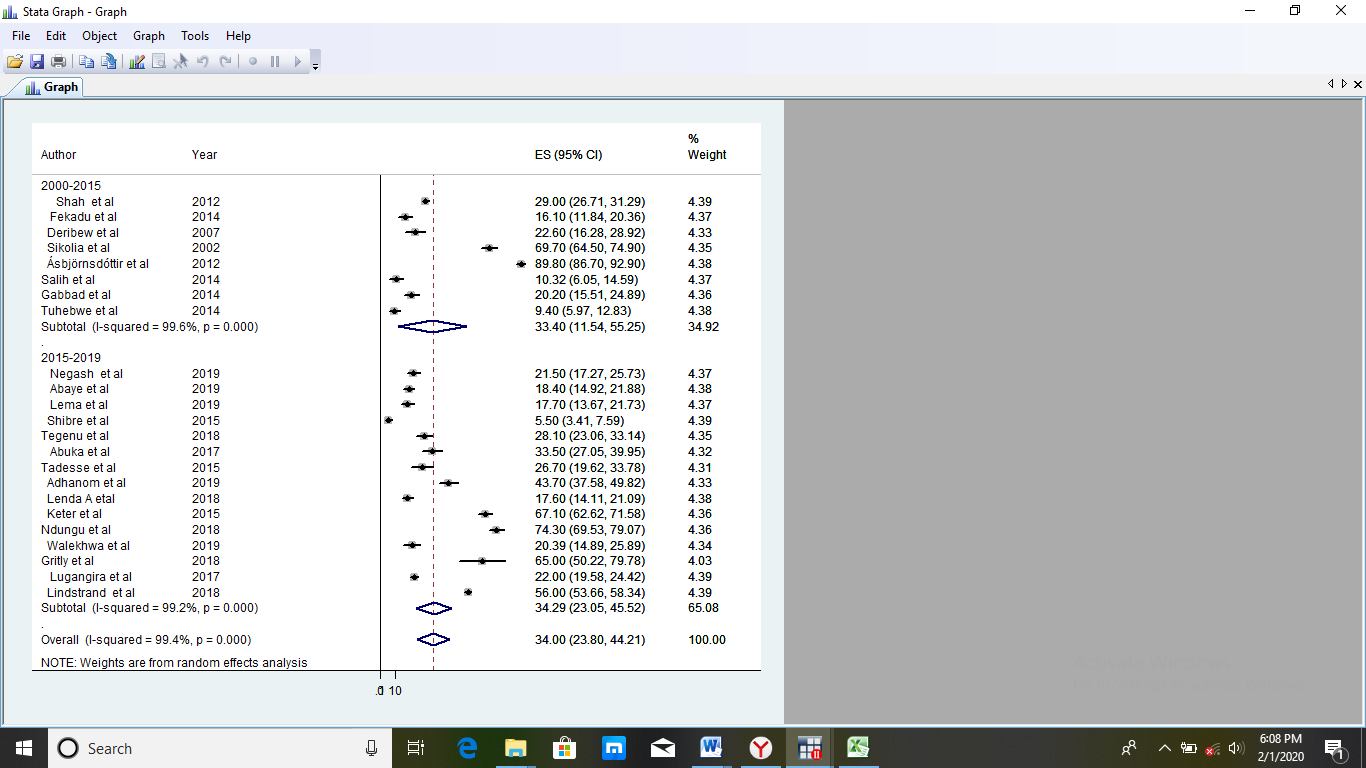


Supplementary Figure 3: Forest plot showing subgroup analysis (by country) of pooled prevalence of pneumonia among under-five children in Ethiopia from2002 up to 2019.


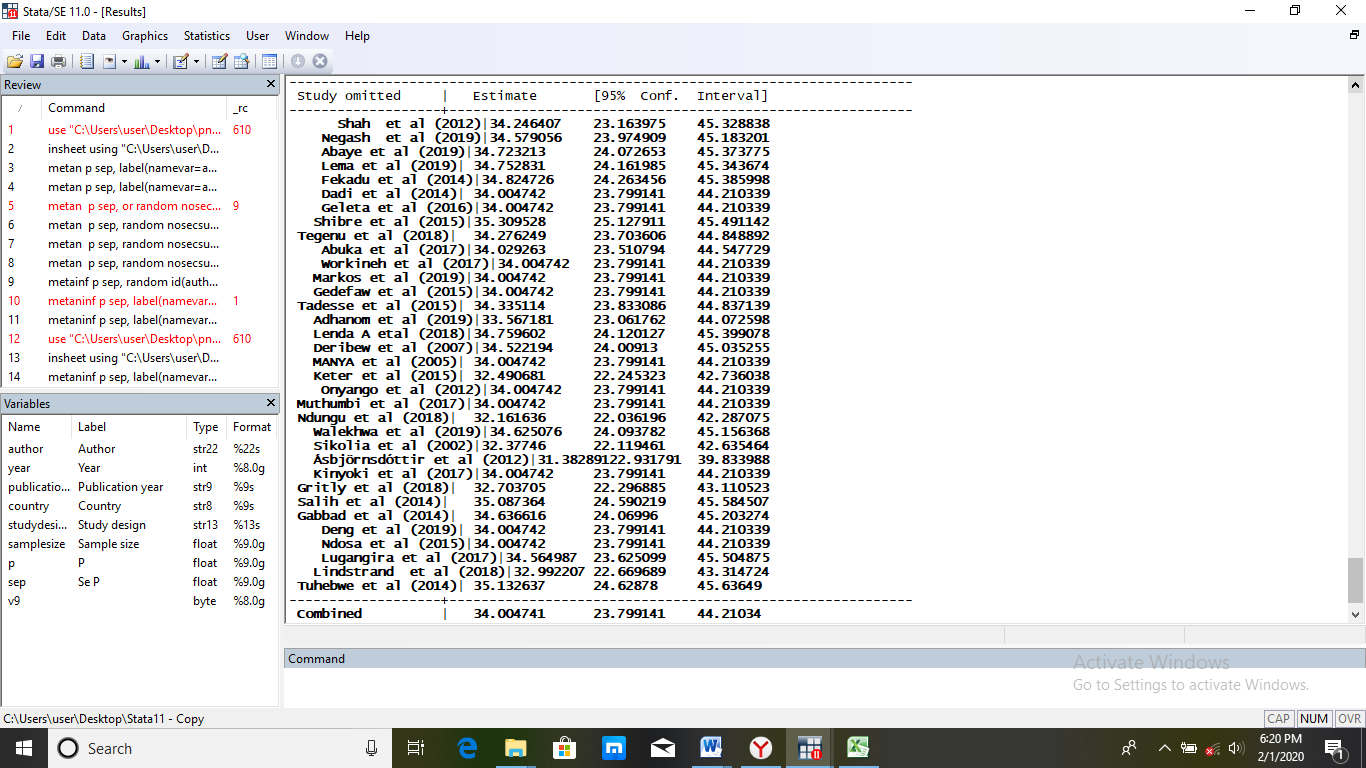


Supplementary Figure 4: sensitivity of pooled prevalence of pneumonia among under-five children in Ethiopia from2002 up to 2019.


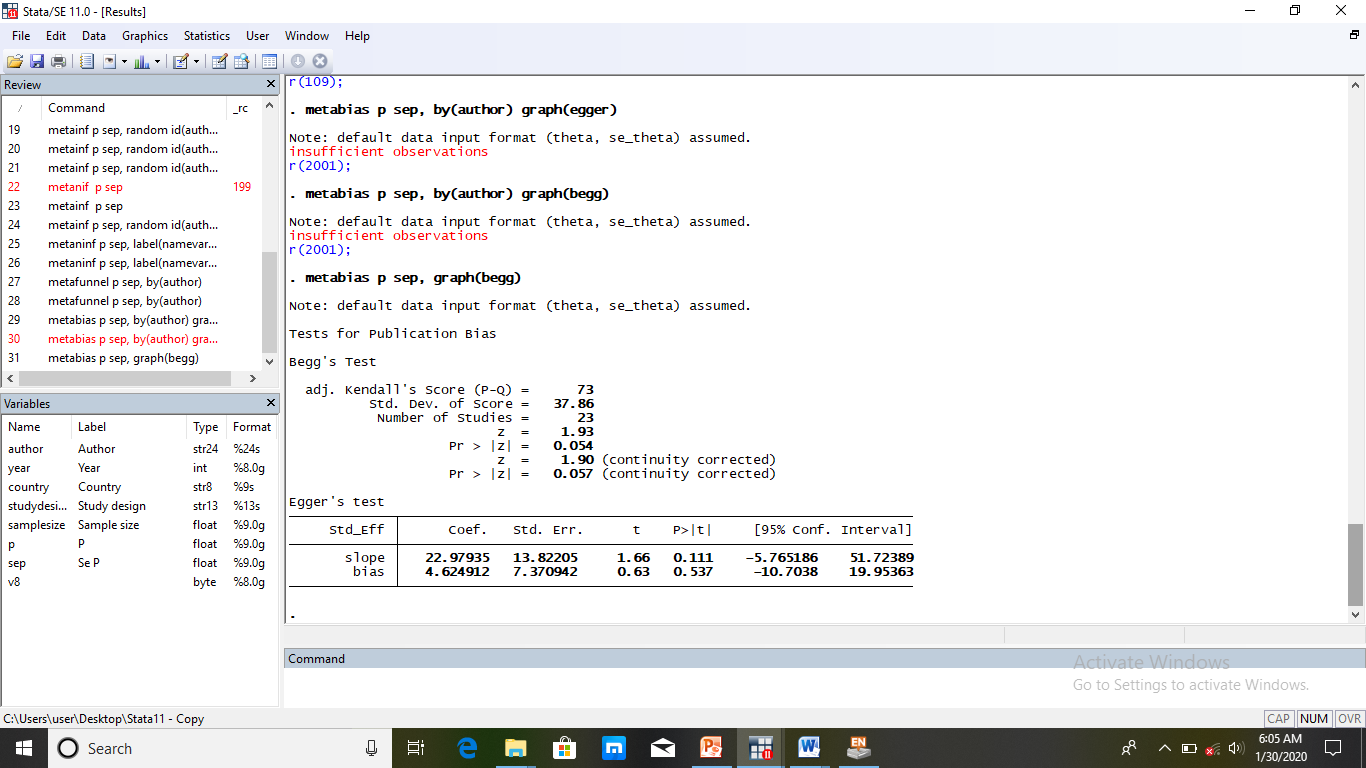


Supplementary Figure 5: publication bias of pooled prevalence of pneumonia among under-five children in Ethiopia from2002 up to 2019.


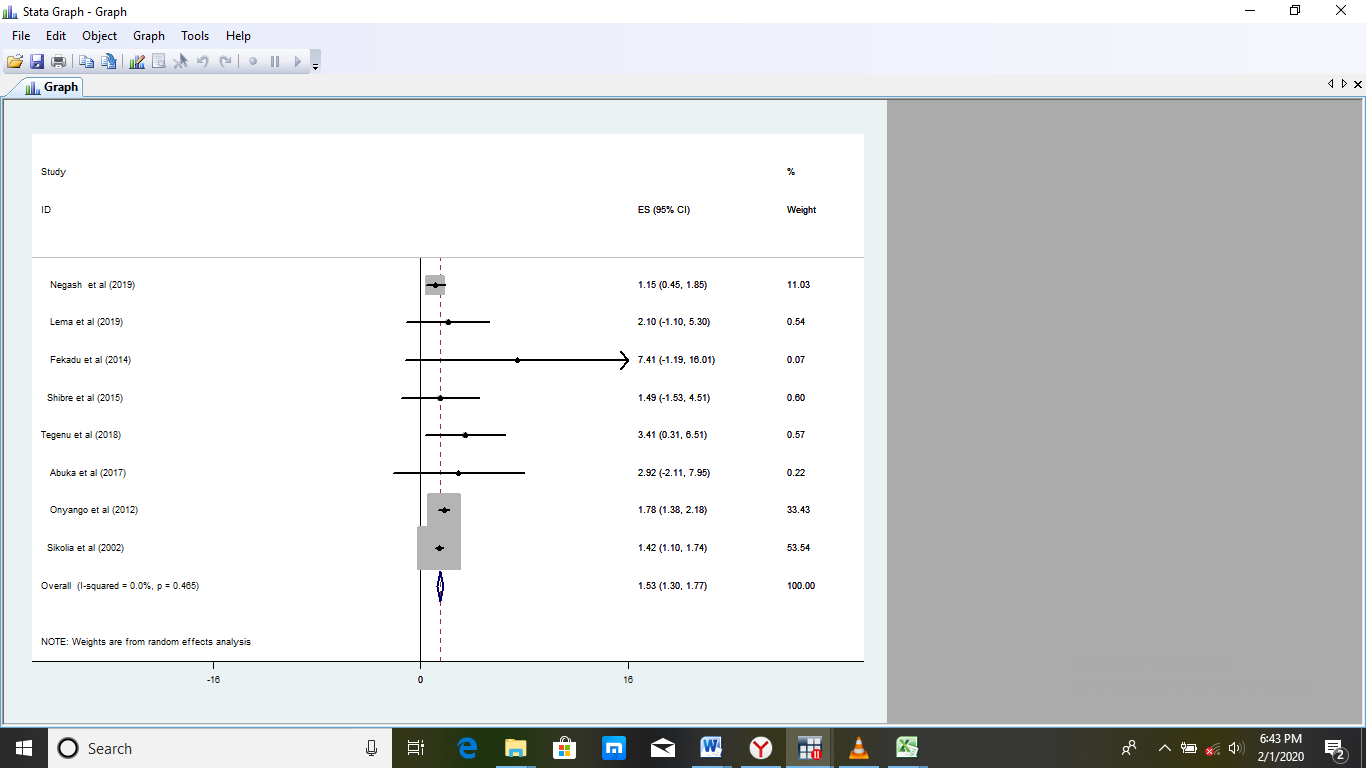


Supplementary Figure 6: Forest plot showing of pooled estimate of AOR for using wood as fuel source as a predictor of pneumonia among under-five children in Ethiopia from2002 up to 2019.


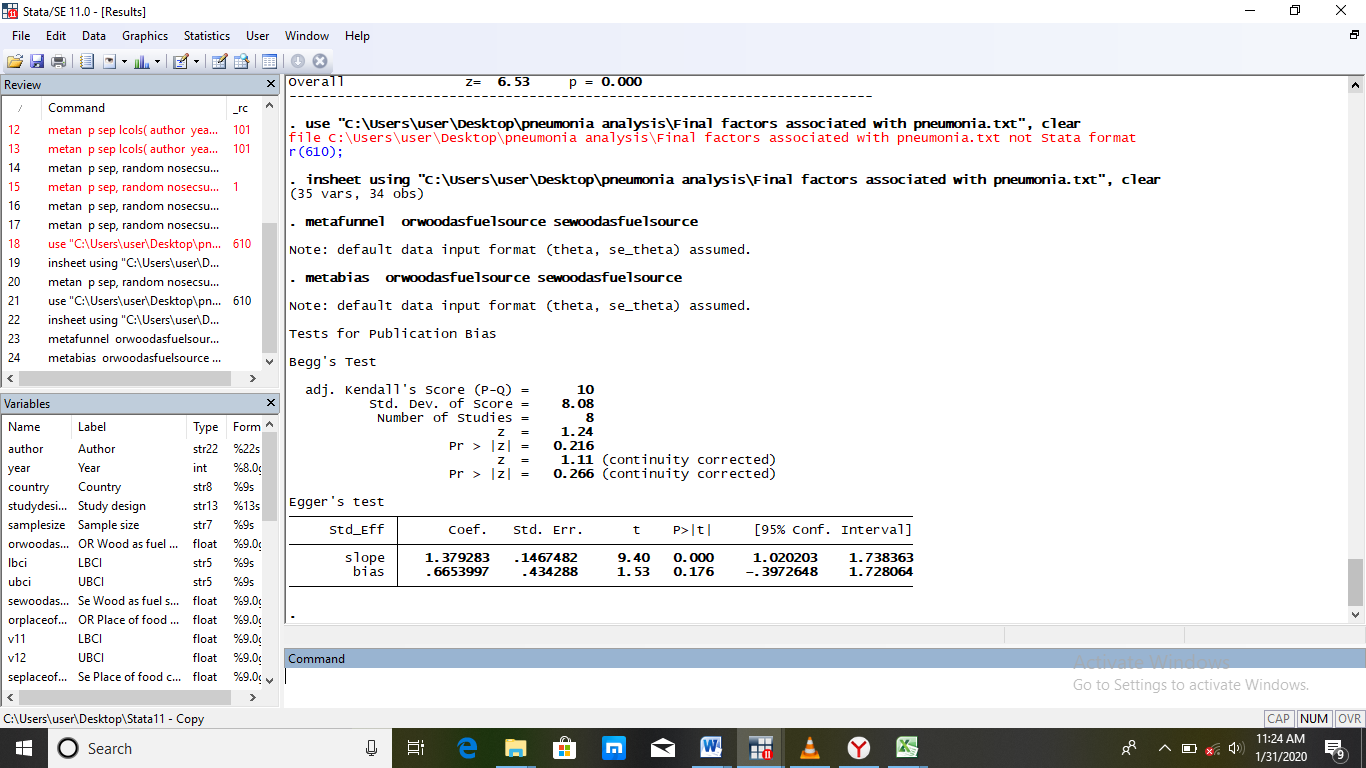


Supplementary Figure 7: publication bias of pooled estimate of AOR for using wood as fuel source as a predictor of pneumonia among under-five children in Ethiopia from2002 up to 2019.


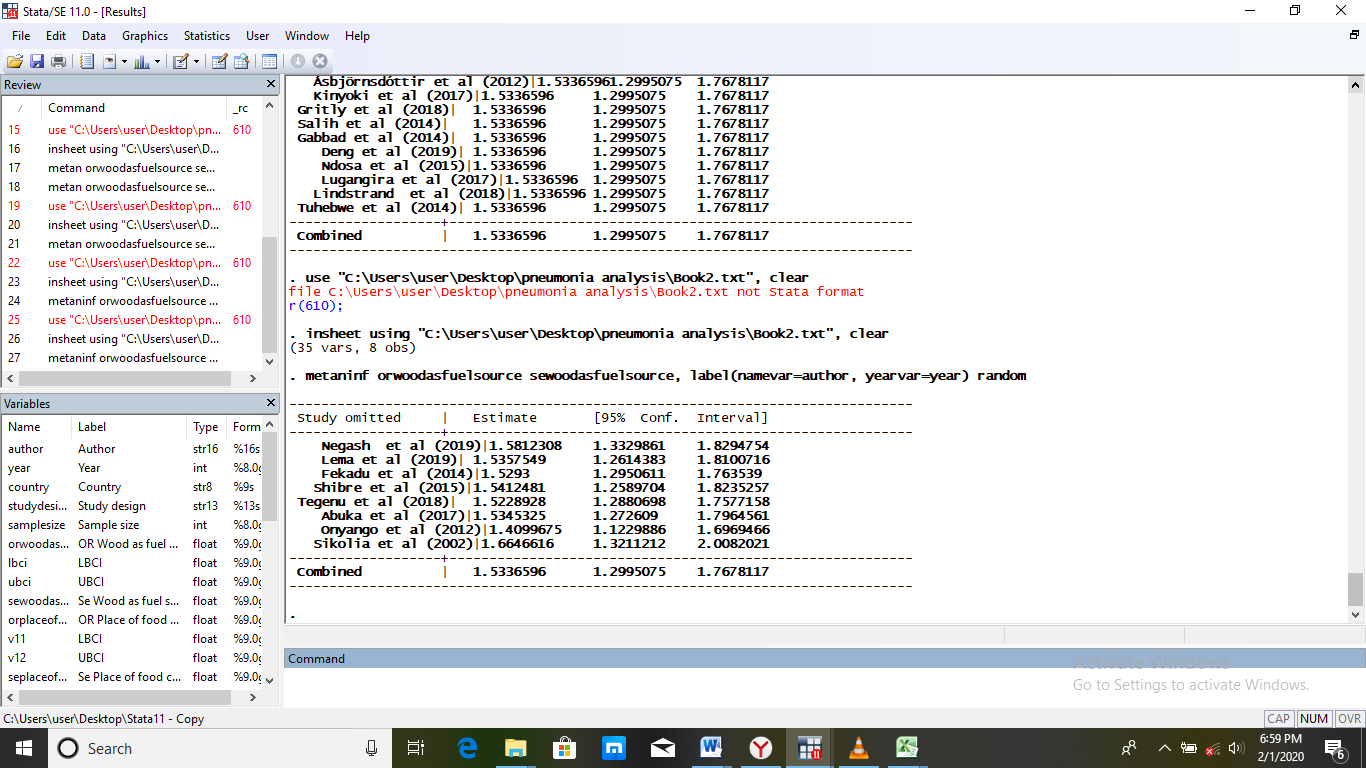


Supplementary Figure 8: sensitivity analysis of pooled estimate of AOR for using wood as fuel source as a predictor of pneumonia among under-five children in Ethiopia from2002 up to 2019.


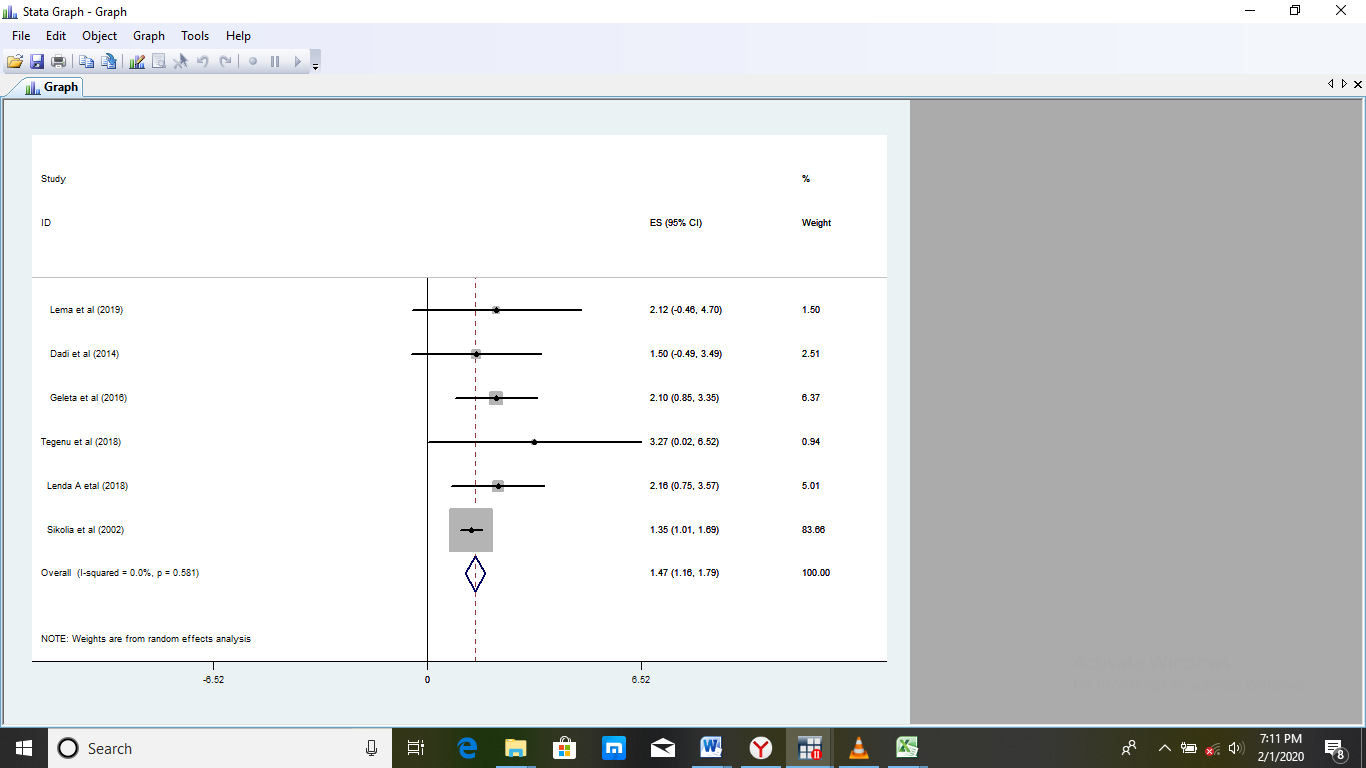


Supplementary Figure 9: Forest plot showing the pooled estimate of AOR for cooking food at home as a predictor of pneumonia among under-five children in Ethiopia from2002 up to 2019.


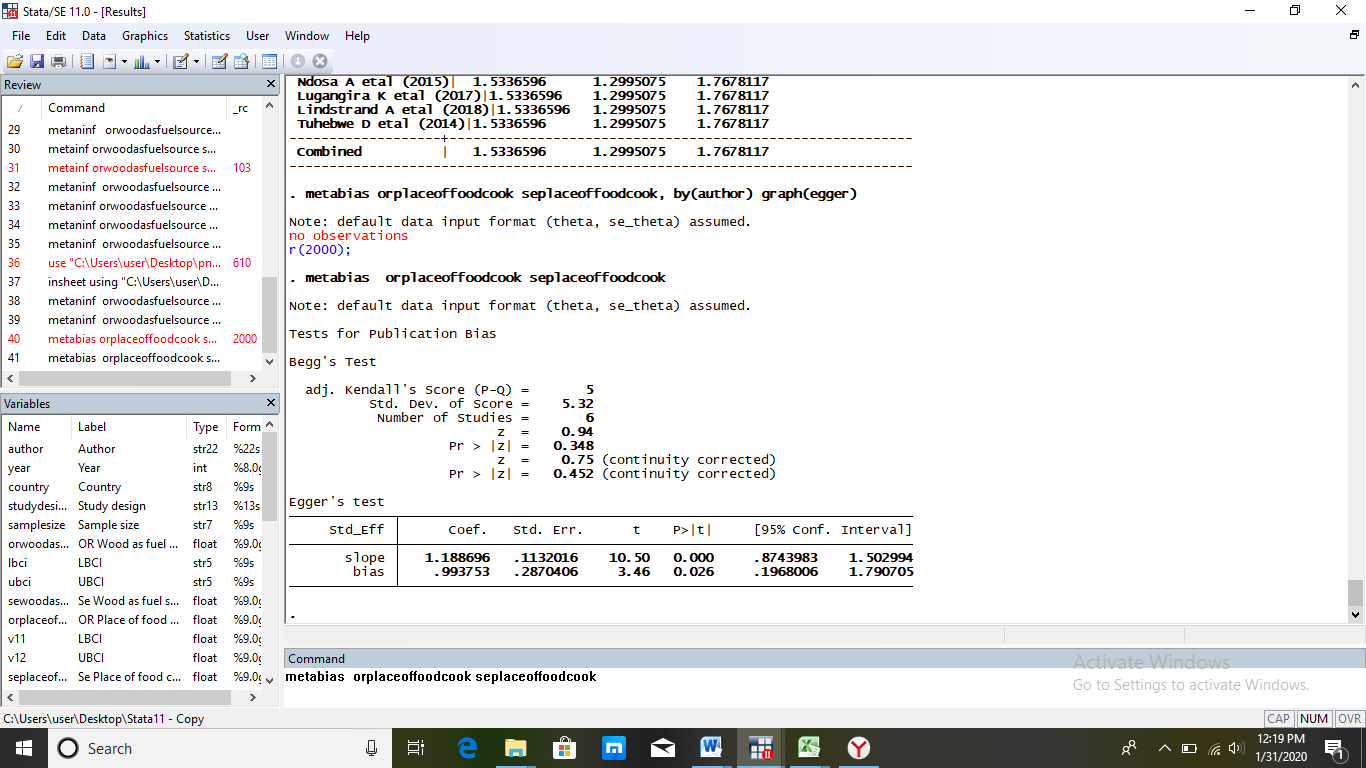


Supplementary Figure 10: publication bias for pooled estimate of AOR for cooking food at home as a predictor of pneumonia among under-five children in Ethiopia from2002 up to 2019.


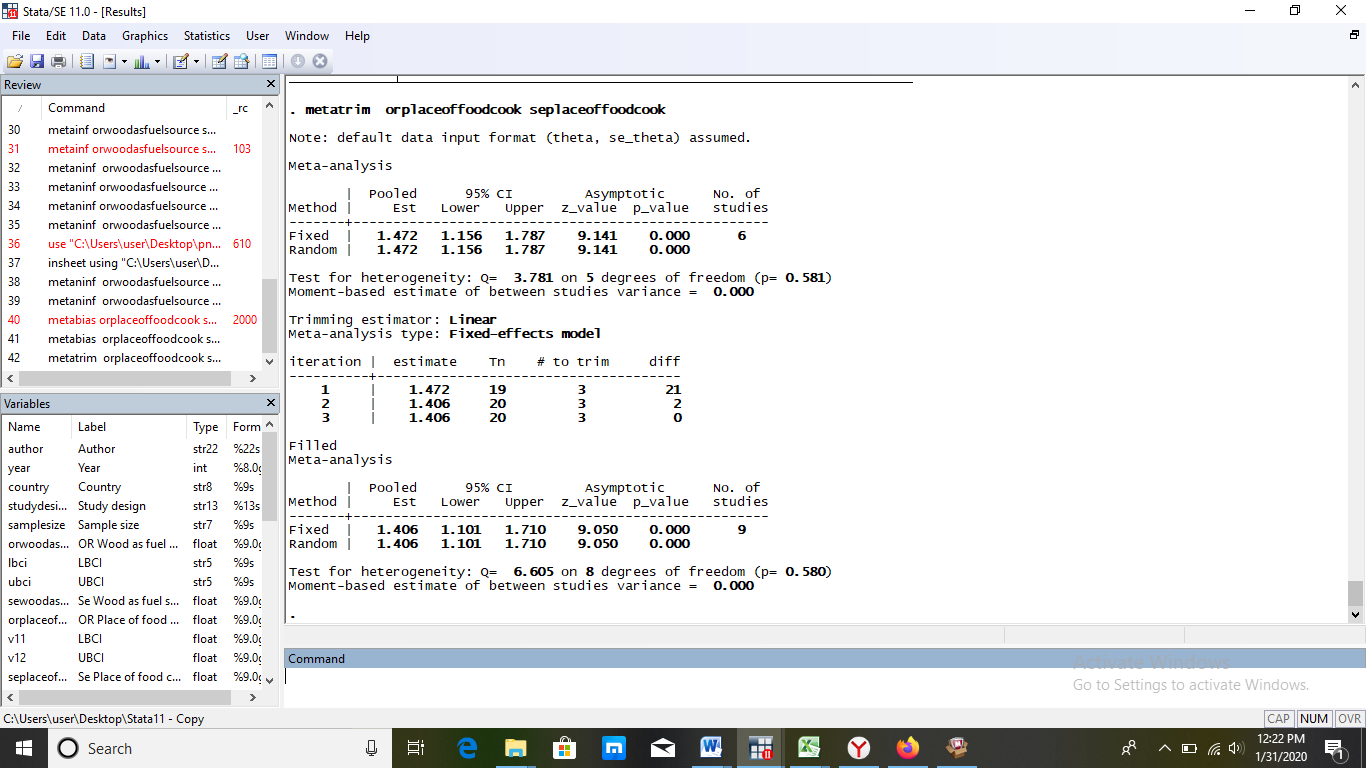


Supplementary Figure 11: Trim and fill analysis for pooled estimate of AOR for cooking food at home as a predictor of pneumonia among under-five children in Ethiopia from2002 up to 2019.


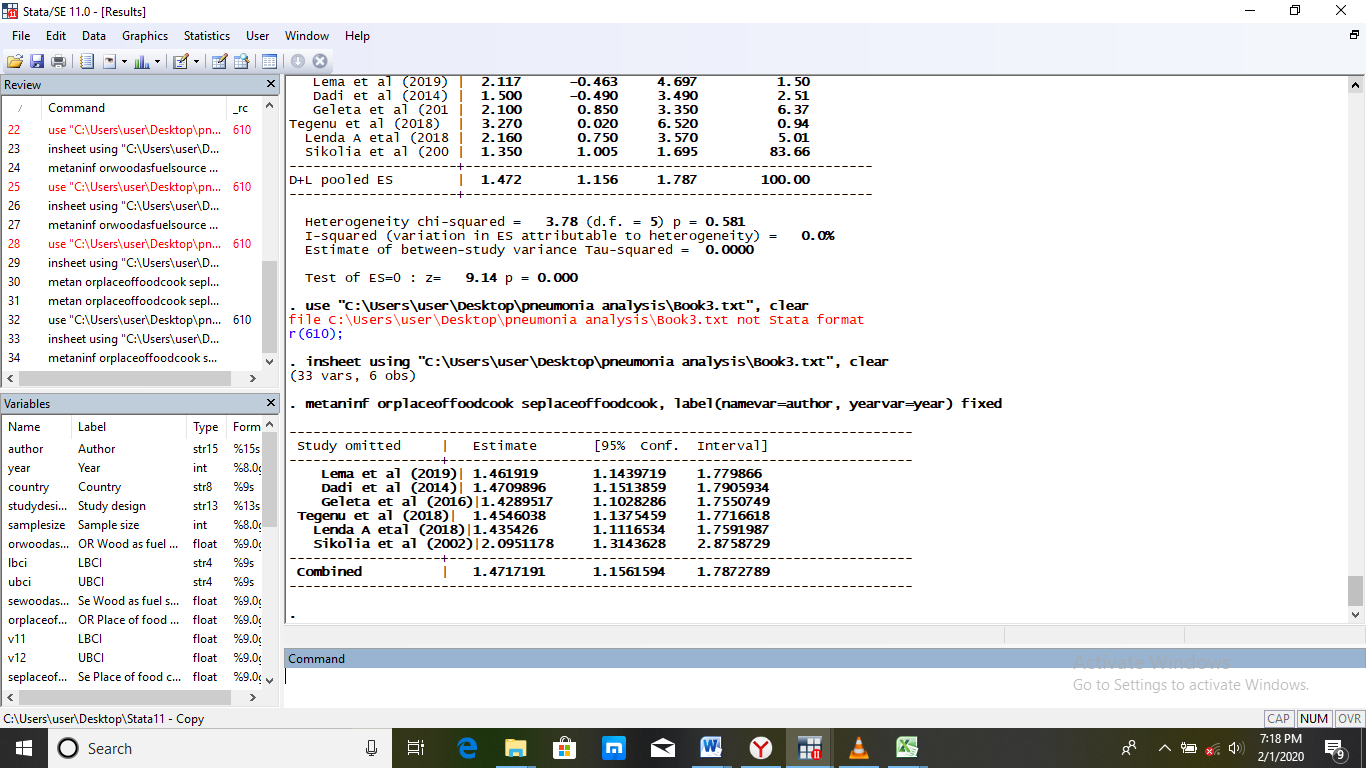


Supplementary Figure 12: Sensitivity analysis for pooled estimate of AOR for cooking food at home as a predictor of pneumonia among under-five children in Ethiopia from2002 up to 2019.


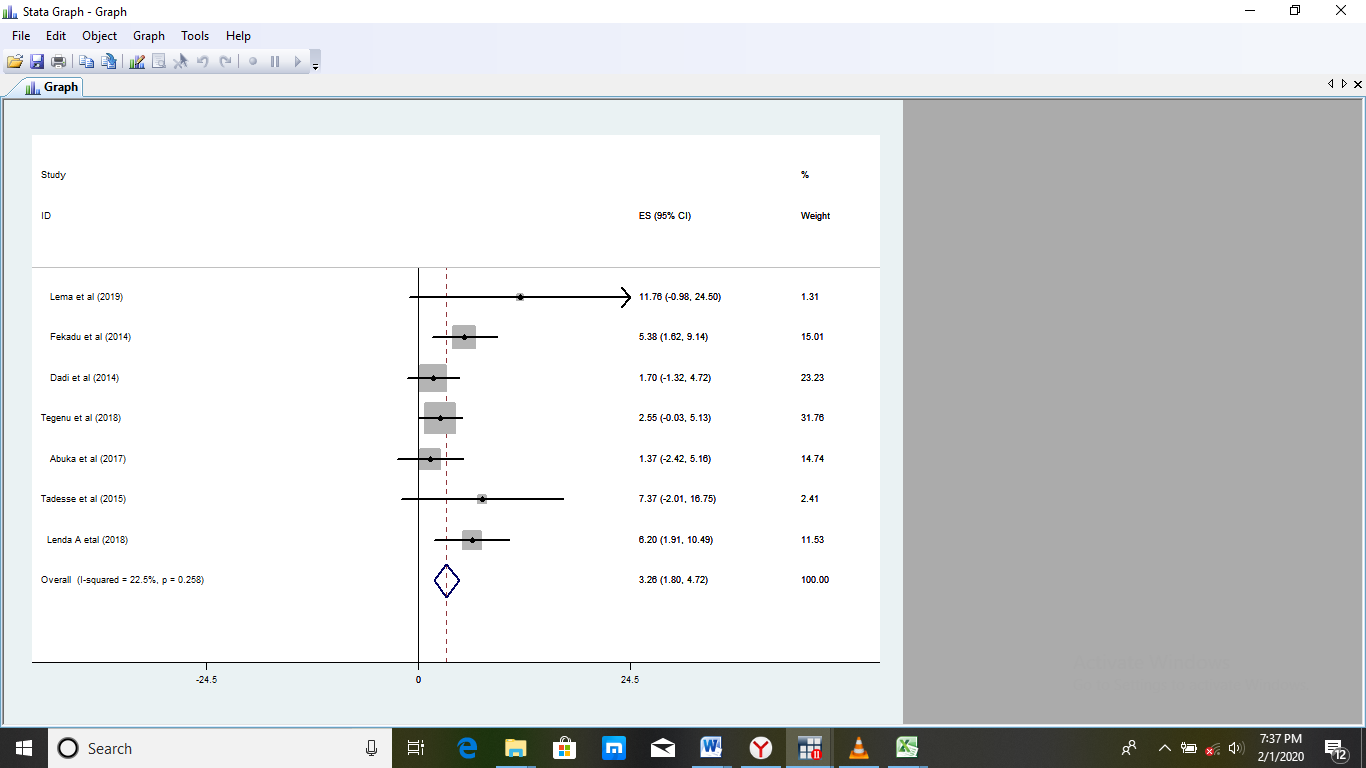


Supplementary Figure 13: Forest plot showing estimate of AOR for caring of the child on mothers during cooking as a predictor of pneumonia among under-five children in Ethiopia from2002 up to 2019.


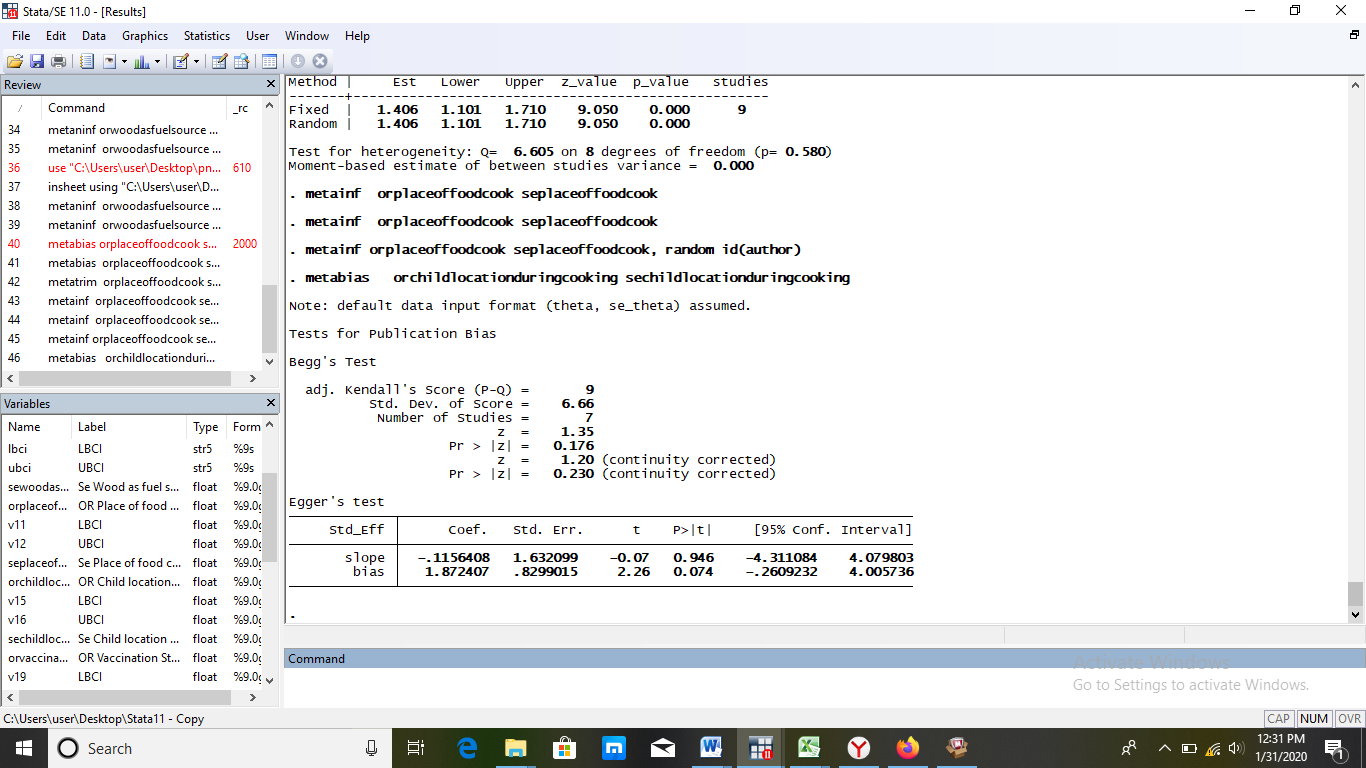


Supplementary Figure 14: publication bias for estimate of AOR for caring of the child on mothers during cooking as a predictor of pneumonia among under-five children in Ethiopia from2002 up to 2019.


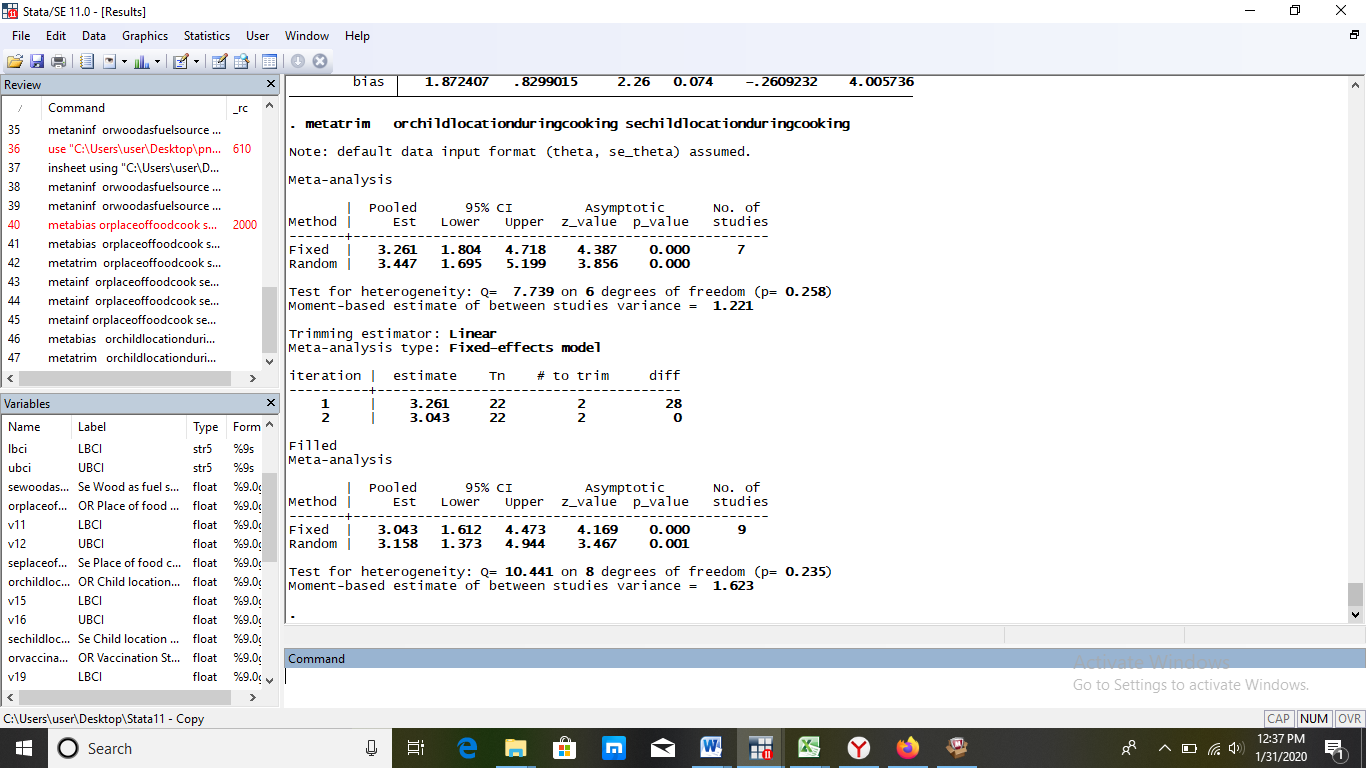


Supplementary Figure 15: trim and fill analysis for estimate of AOR for caring of the child on mothers during cooking as a predictor of pneumonia among under-five children in Ethiopia from2002 up to 2019.


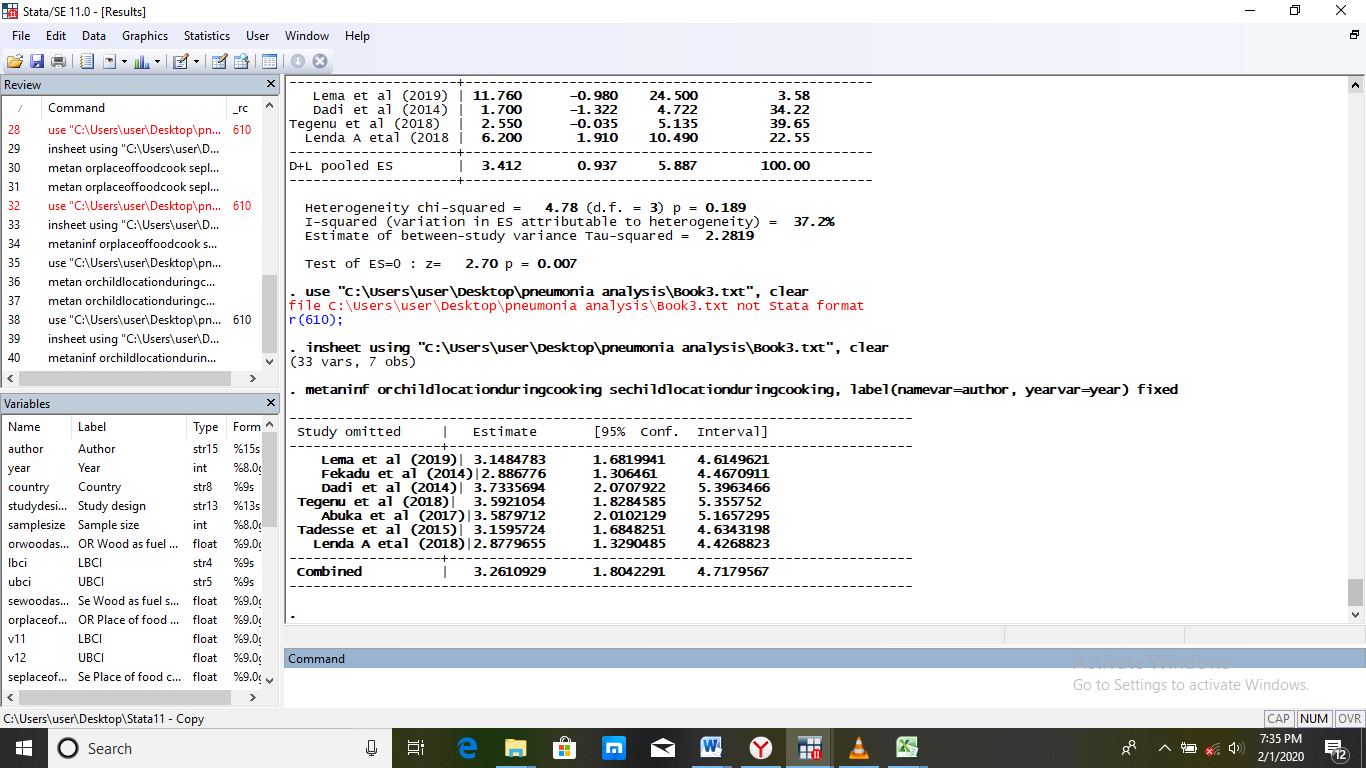


Supplementary Figure 16: sensitivity analysis for estimate of AOR for caring of the child on mothers during cooking as a predictor of pneumonia among under-five children in Ethiopia from2002 up to 2019.


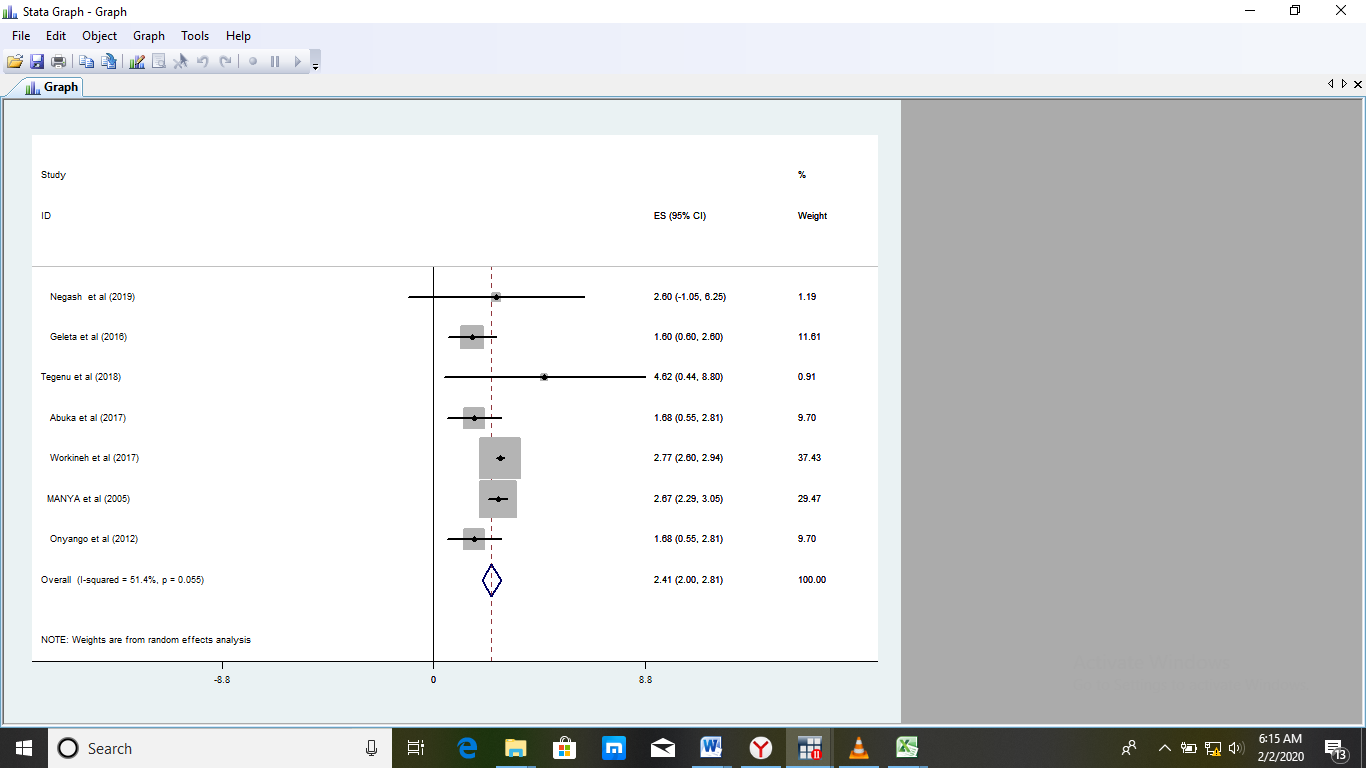


Supplementary Figure 17: Forest plot showing the pooled estimate of AOR for being unvaccinated as a predictor of pneumonia among under-five children in Ethiopia from2002 up to 2019.


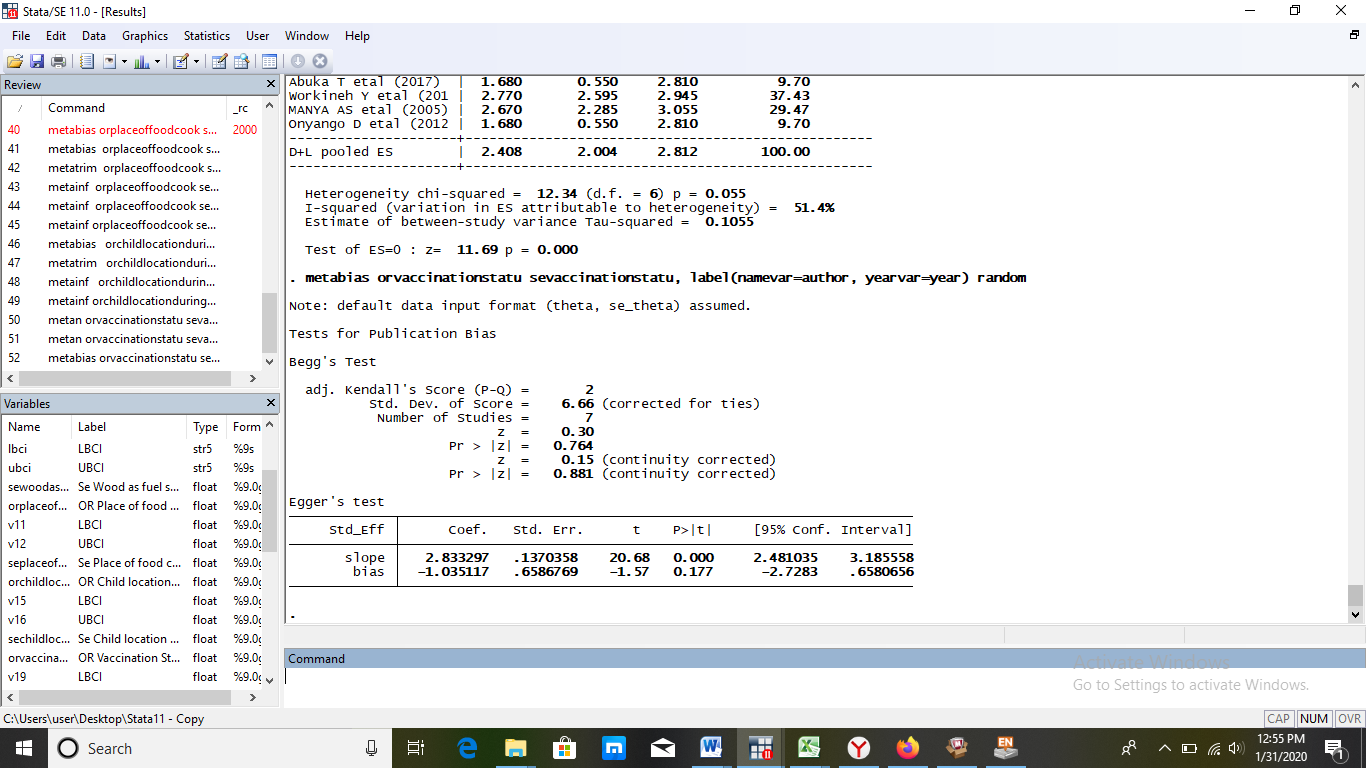


Supplementary Figure 18: publication bias for pooled estimate of AOR for being unvaccinated as a predictor of pneumonia among under-five children in Ethiopia from2002 up to 2019.


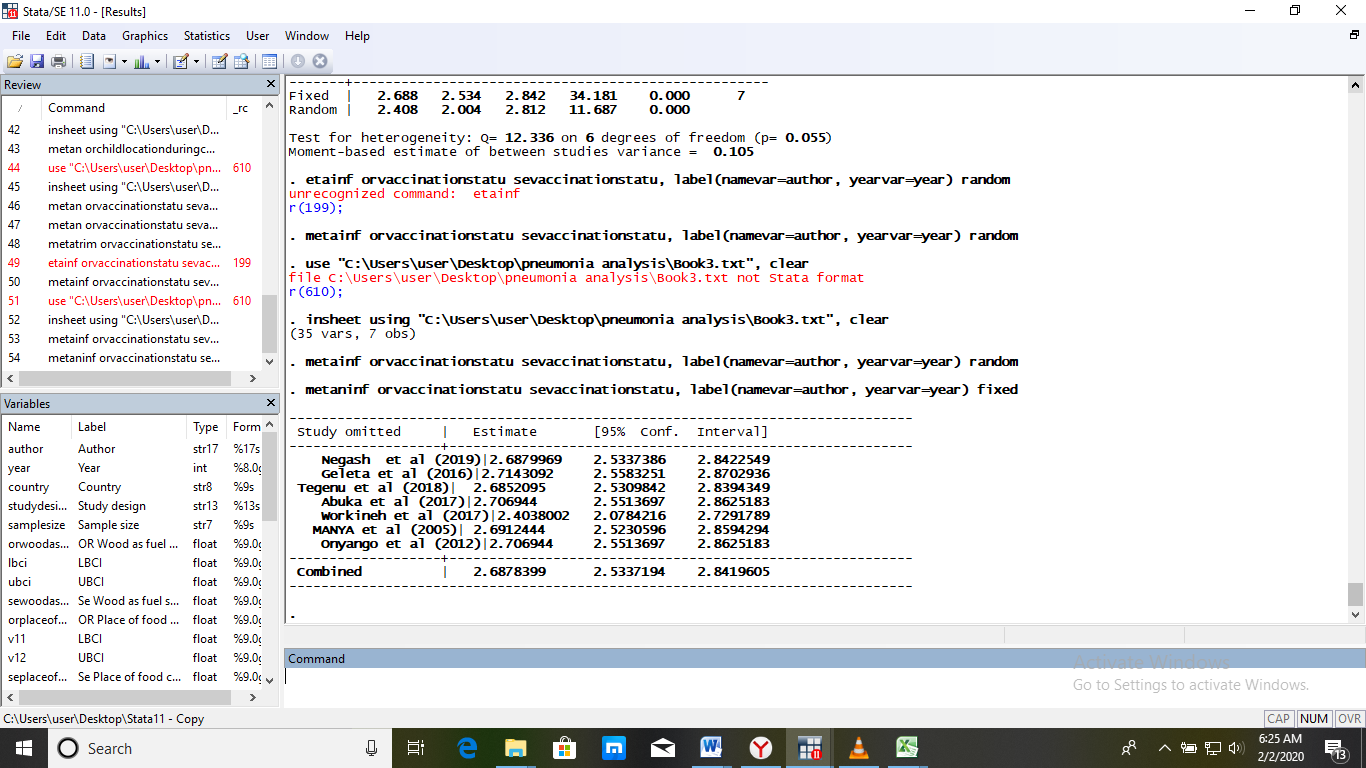


Supplementary Figure 19: sensitivity analysis for pooled estimate of AOR for being unvaccinated as a predictor of pneumonia among under-five children in Ethiopia from2002 up to 2019.


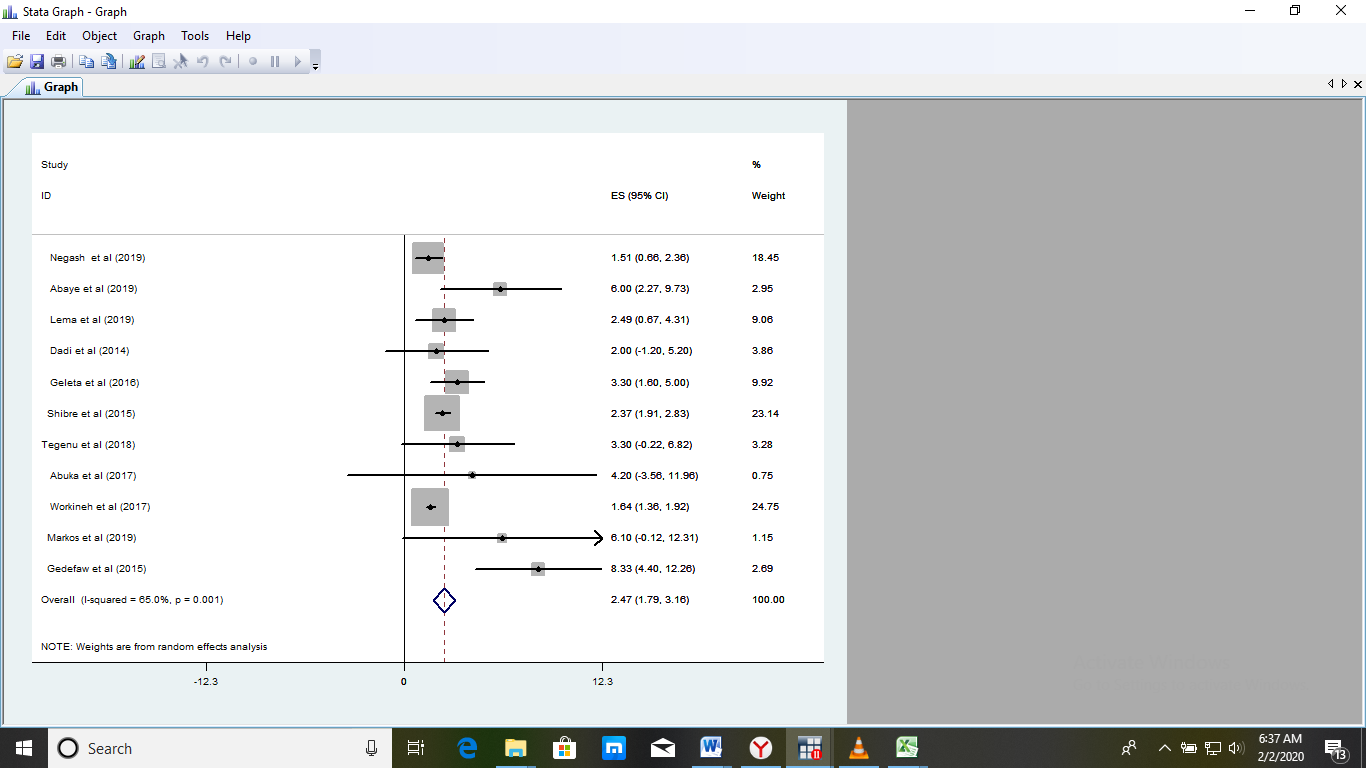


Supplementary Figure 20:Forest plot showing the pooled estimate of AOR for non-exclusive breast feeding as a predictor of pneumonia among under-five children in Ethiopia from 2002 up to 2019.


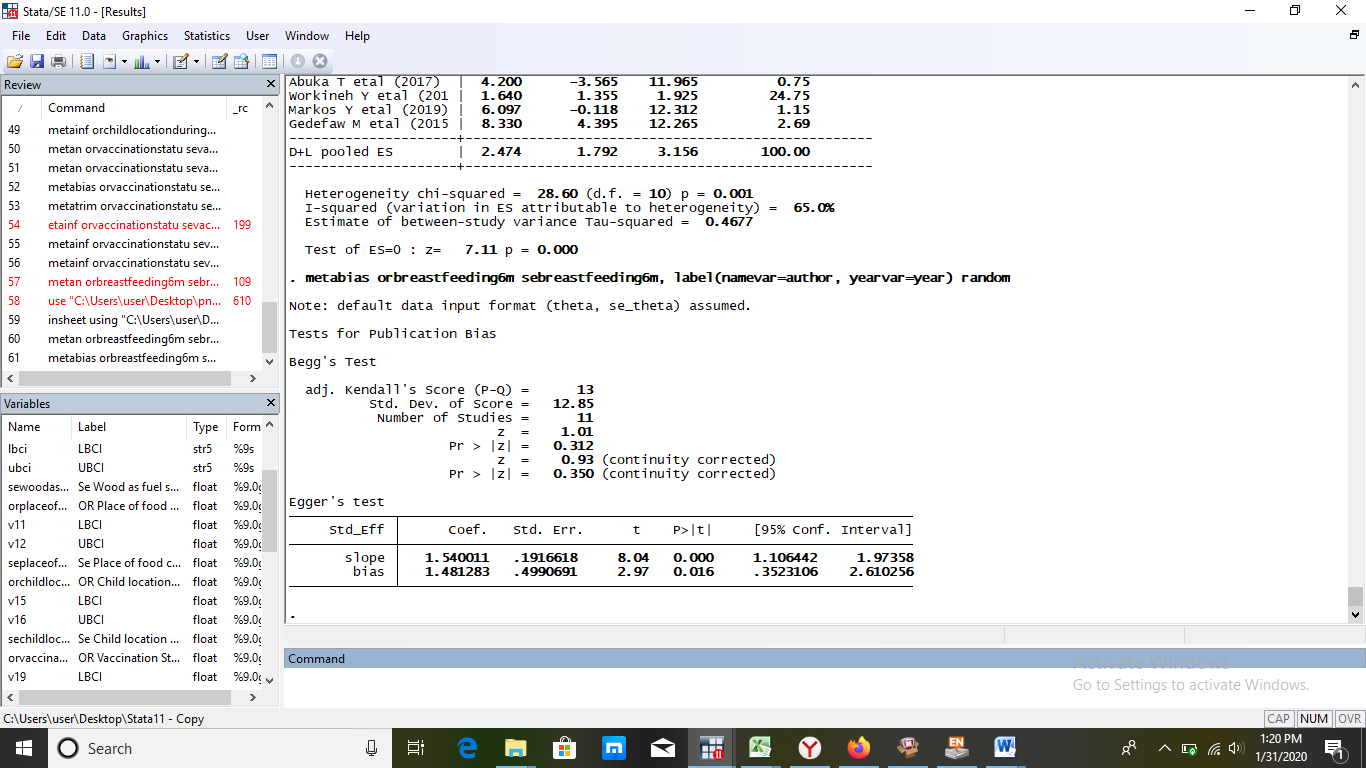


Supplementary Figure 21: publication bias for the pooled estimate of AOR for non-exclusive breast feeding as a predictor of pneumonia among under-five children in Ethiopia from 2002 up to 2019.


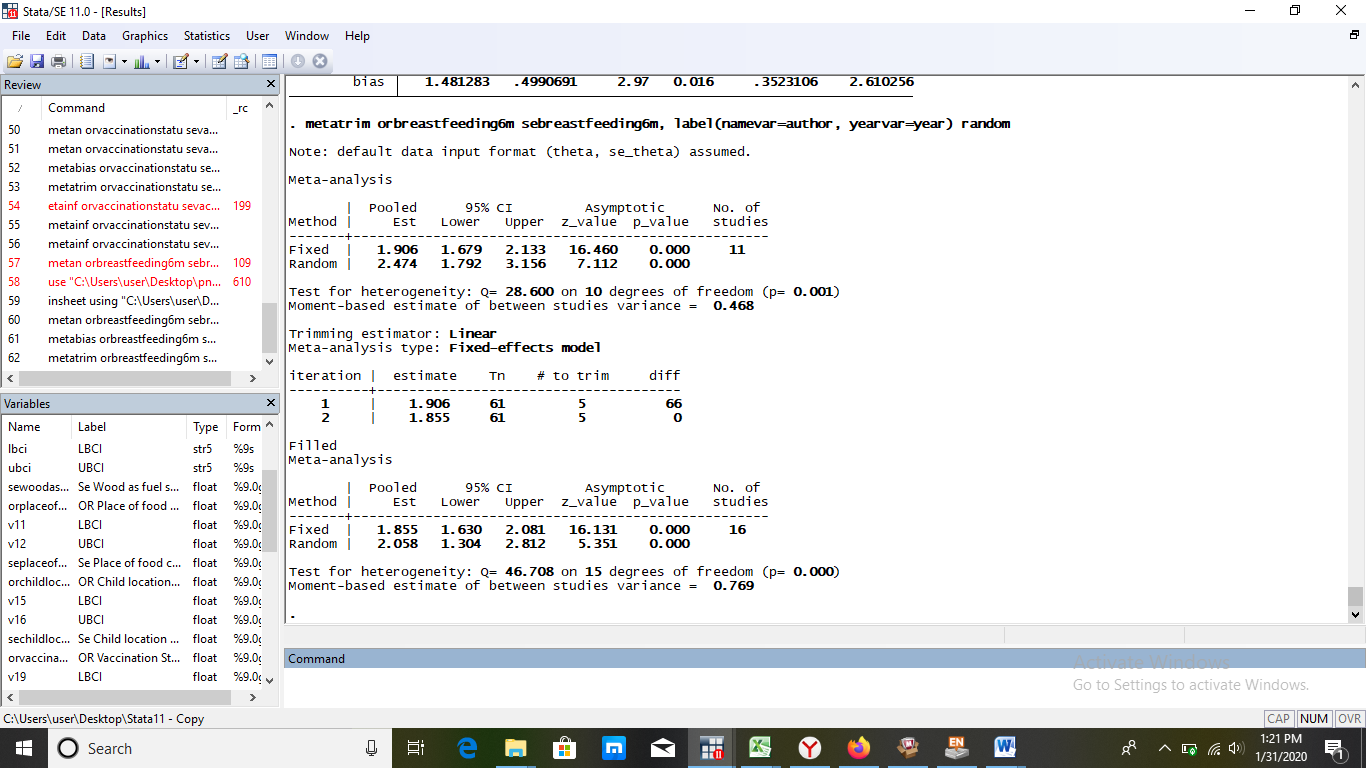


Supplementary Figure 22: Trim and fill analysis for the pooled estimate of AOR for non-exclusive breast feeding as a predictor of pneumonia among under-five children in Ethiopia from 2002 up to 2019.


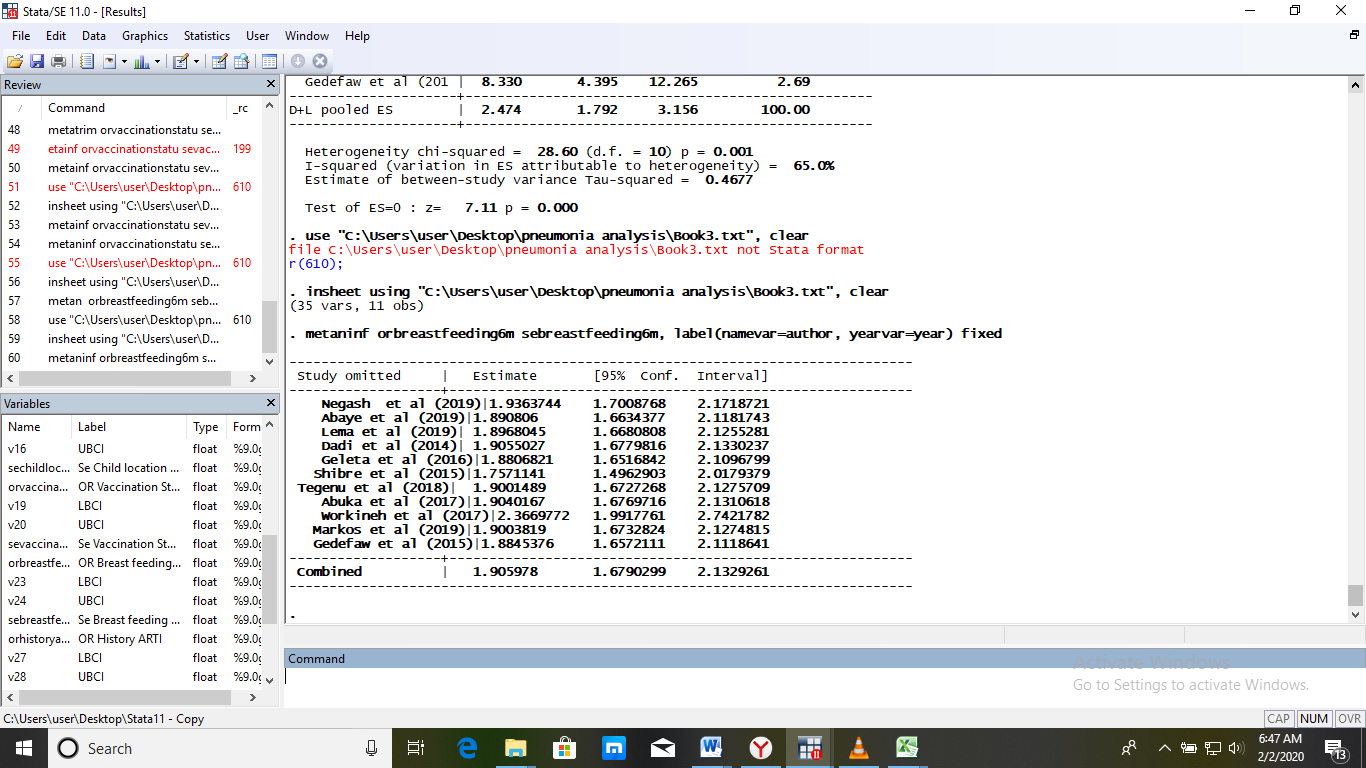


Supplementary Figure 23: sensitivity analysis for the pooled estimate of AOR for non-exclusive breast feeding as a predictor of pneumonia among under-five children in Ethiopia from 2002 up to 2019.


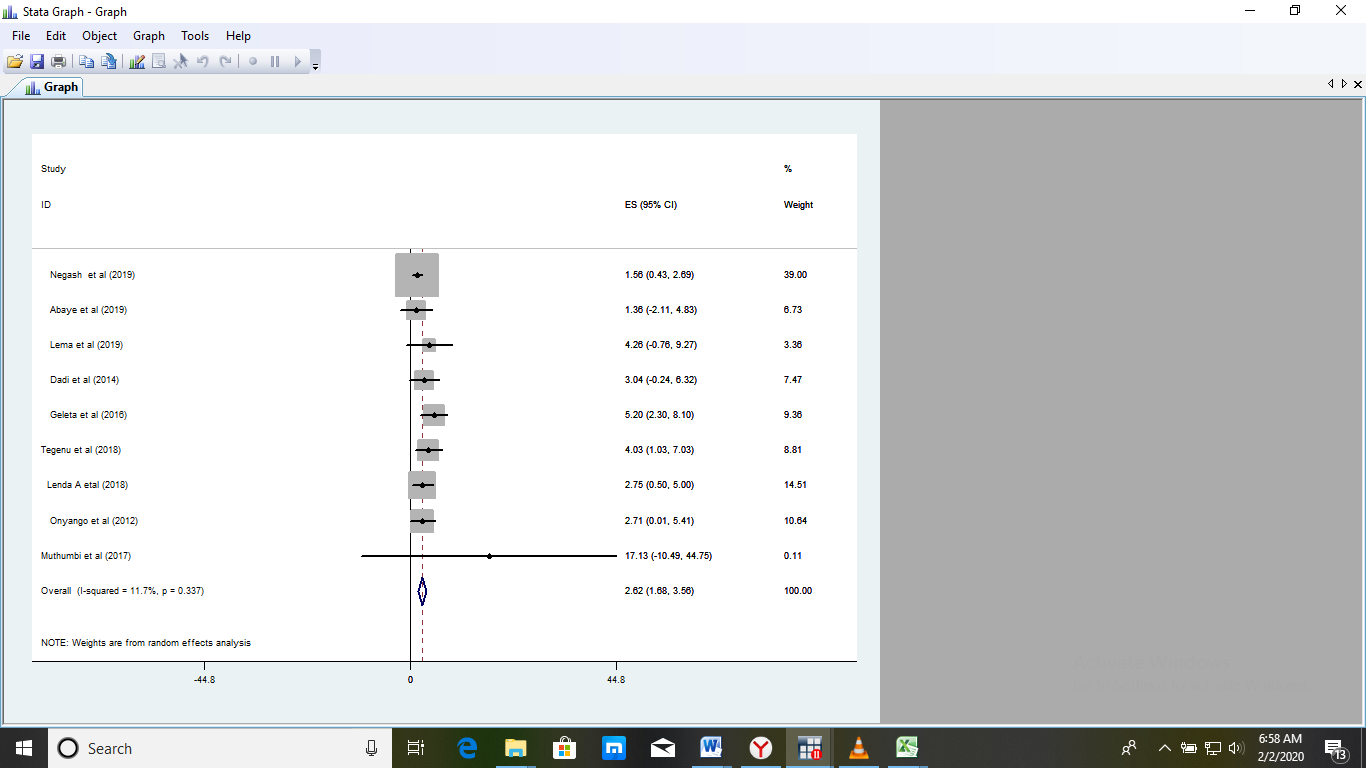


Supplementary Figure 24: Forest plot showing the pooled estimate of AOR for history of ARTI as a predictor of pneumonia among under-five children in Ethiopia from 2002 up to 2019.


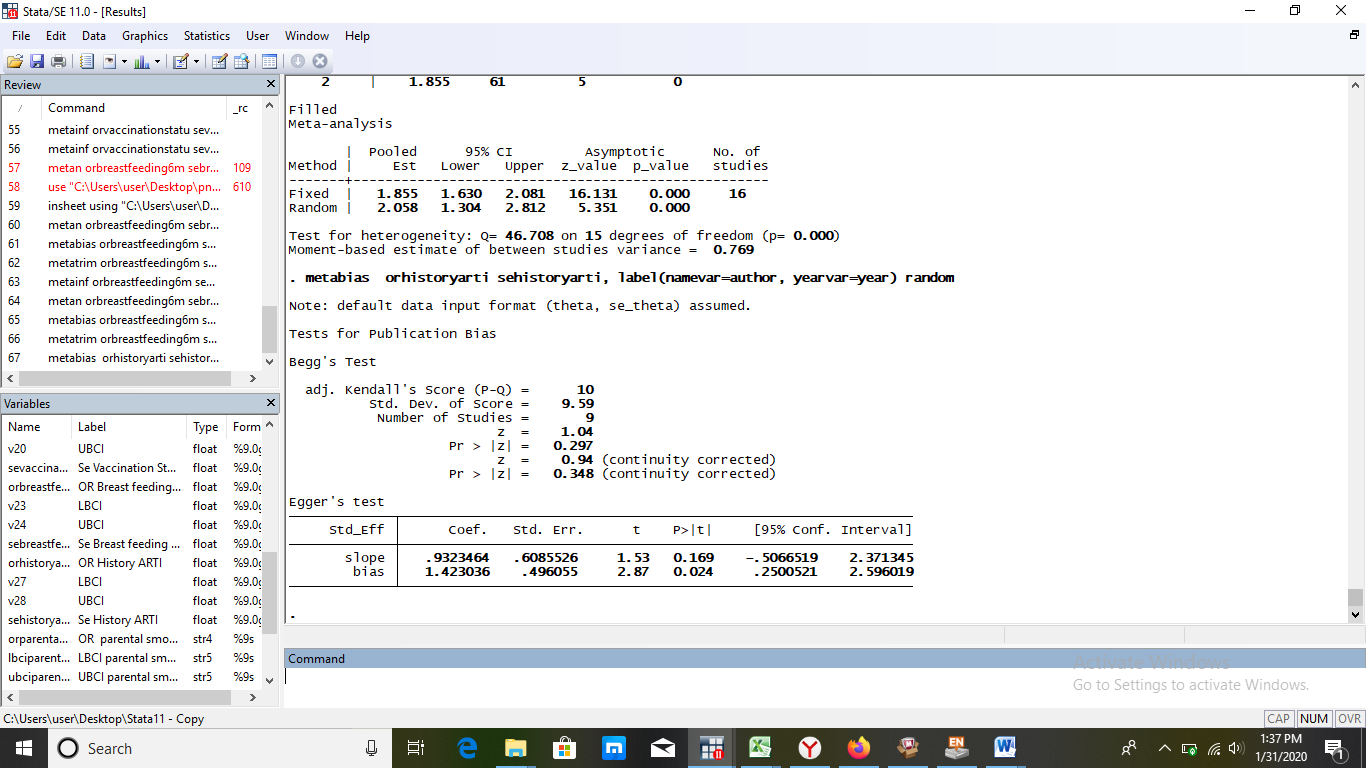


Supplementary Figure 25: Publication bias for the pooled estimate of AOR for history of ARTI as a predictor of pneumonia among under-five children in Ethiopia from 2002 up to 2019.


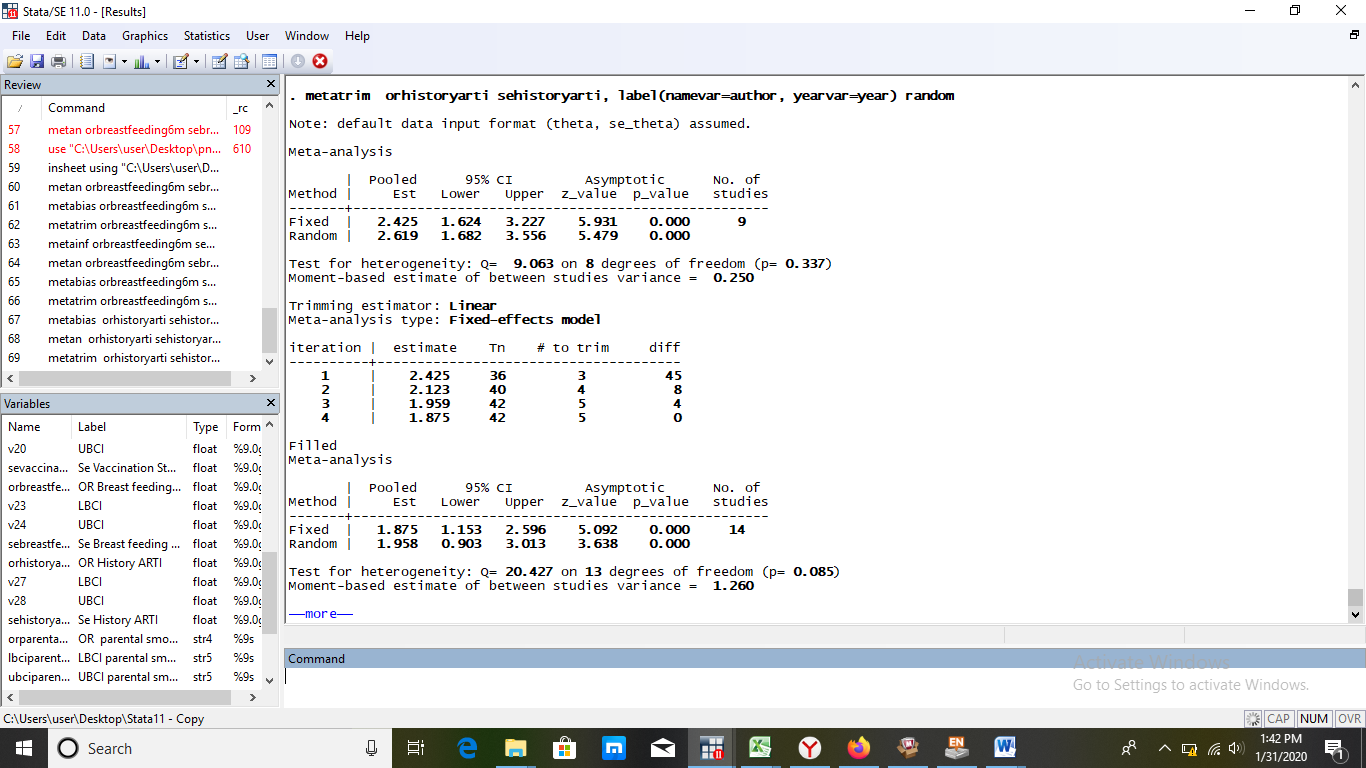


Supplementary Figure 26: Trim and fill analysis for the pooled estimate of AOR for history of ARTI as a predictor of pneumonia among under-five children in Ethiopia from 2002 up to 2019.


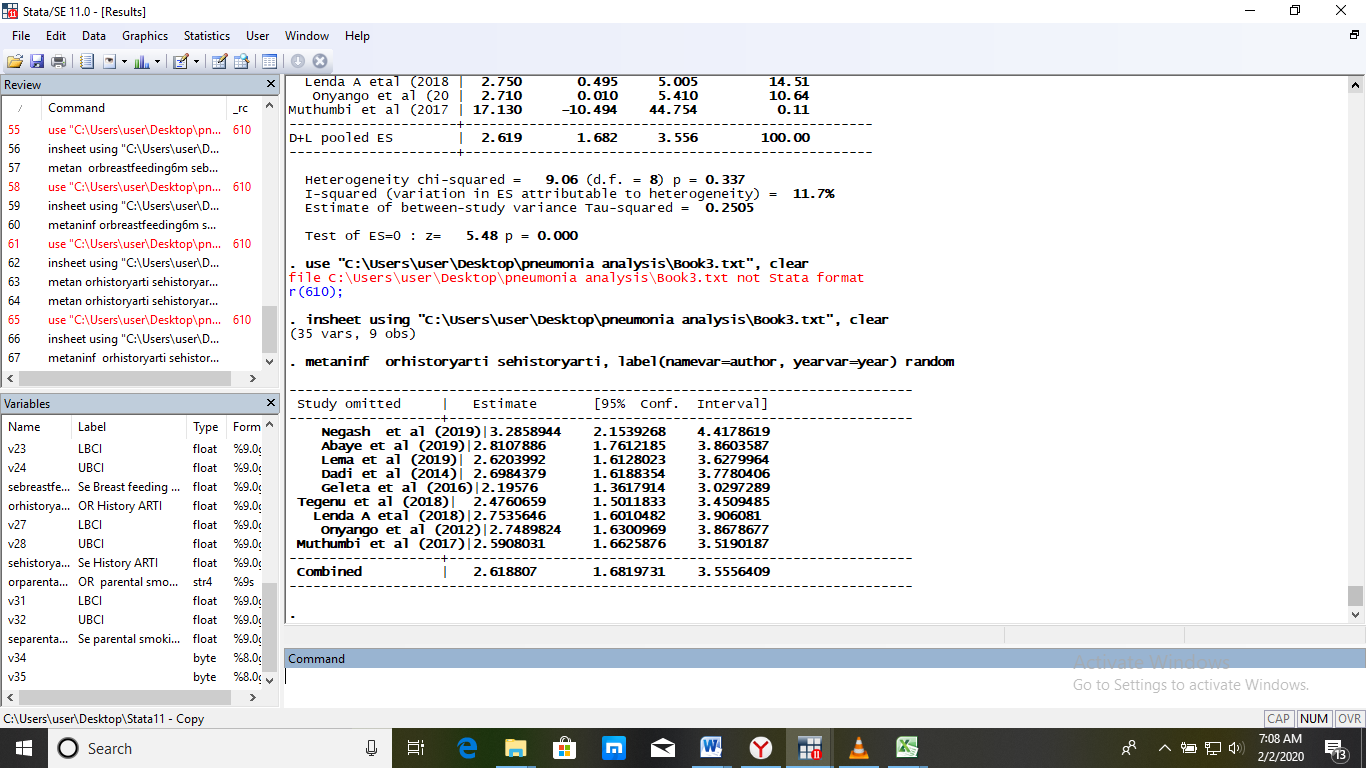


Supplementary Figure 27: sensitivity analysis for the pooled estimate of AOR for history of ARTI as a predictor of pneumonia among under-five children in Ethiopia from 2002 up to 2019

**References**

1. Shah S, Zemichael O, Meng HD. Factors associated with mortality and length of stay in hospitalised neonates in Eritrea, Africa: a cross-sectional study. BMJ open. 2012;2(5):e000792.

2. Tadesse R. Household Biomass Fuel Use and Acute Respiratory Infections among Younger Children: An Exposure Assessment in Anilemo Woreda, Southern Ethiopia: Addis Ababa University; 2015.

3. Abaye G, Fekadu H, Haji K, Alemu D, Anjulo AA, Yadate DT. Prevalence and risk factors of pneumococcal nasopharyngeal carriage in healthy children attending kindergarten, in district of Arsi Zone, South East, Ethiopia. BMC research notes. 2019;12(1):253.

4. Lema B, Seyoum K, Atlaw D. Prevalence of Community Acquired Pneumonia among Children 2 to 59 Months Old and its Associated Factors in Munesa District, Arsi Zone, Oromia Region, Ethiopia. Clinics Mother Child Health. 2019;16:334.

5. Fekadu GA, Terefe MW, Alemie GA. Prevalence of pneumonia among under-five children in Este Town and the surrounding rural Kebeles, Northwest Ethiopia: a community based cross sectional study. Science Journal of Public Health. 2014;2(3):150-5.

6. Dadi AF, Kebede Y, Birhanu Z. Determinants of pneumonia in children aged two months to five years in urban areas of Oromia Zone, Amhara Region, Ethiopia. Open Access Library Journal. 2014;1(08):1.

7. Geleta D, Tessema F, Ewnetu H. Determinants of community acquired pneumonia among children in Kersa District, Southwest Ethiopia: facility based case control study. J Pediatr Neonatal Care. 2016;5(2):00179.

8. Shibre G. Assessment of the Prevalence and Associated Factors of Pneumonia in Children 2to 59 Months Old, Debreberhan District, North East Ethiopia: Addis Abeba University; 2015.

9. Tegenu K. Prevalence and associated factors of pneumonia among under-five children at public hospitals in Jimma zone, South West of Ethiopia, 2018: Addis Ababa Universty; 2018.

10. Abuka T. Prevalence of pneumonia and factors associated among children 2-59 months old in Wondo Genet district, Sidama zone, SNNPR, Ethiopia. Current Pediatric Research. 2017.

11. Adhanom G, Gebreegziabiher D, Weldu Y, Gebreyesus Wasihun A, Araya T, Legese H, et al. Species, Risk Factors, and Antimicrobial Susceptibility Profiles of Bacterial Isolates from HIV-Infected Patients Suspected to Have Pneumonia in Mekelle Zone, Tigray, Northern Ethiopia. BioMed research international. 2019;2019.

12. Lenda A, Demena M, Mengistie B. Prevalence of Pneumonia and Associated Factors Among Under-five Children in Boloso Bombe Woreda, Southern Ethiopia: A Community Based Study: Haramaya University; 2018.

13. Keter PKK. Knowledge, Attitudes and Practices of Mothers in relation to Childhood Pneumonia and factors associated with Pneumonia and Seeking Health Care in Kapsabet District Hospital in Nandi County, Kenya: JKUAT; 2015.

14. Muthumbi E, Lowe BS, Muyodi C, Getambu E, Gleeson F, Scott JAG. Risk factors for community-acquired pneumonia among adults in Kenya: a case–control study. Pneumonia. 2017;9(1):17.

15. Ndungu EW, Okwara FN, Oyore JP. Cross Sectional Survey of Care Seeking For Acute Respiratory Illness in Children Under 5 Years in Rural Kenya. Am J Pediatr. 2018;4(3):69-79.

16. Walekhwa M, Muturi M, Revathi Gunturu EK, Kabera B. Streptococcus pneumoniae serotype epidemiology among PCV-10 vaccinated and unvaccinated children at Gertrude’s Children’s Hospital, Nairobi County: a cross-sectional study. F1000Research. 2018;7.

17. Kinyoki DK, Manda SO, Moloney GM, Odundo EO, Berkley JA, Noor AM, et al. Modelling the ecological comorbidity of acute respiratory infection, diarrhoea and stunting among children under the age of 5 years in Somalia. International Statistical Review. 2017;85(1):164-76.

18. Gritly SM, Elamin MO, Rahimtullah H, Ali AYH, Dhiblaw A, Mohamed EA, et al. Risk factors of pneumonia among children under 5 years at a pediatric hospital in Sudan. International Journal of Medical Research & Health Sciences. 2018;7(4):60-8.

19. Salih KEM, Bilal JA, Alfadeel MA, Hamid Y, Eldouch W, Elsammani E, et al. Poor adherence to the World Health Organization guidelines of treatment of severe pneumonia in children at Khartoum, Sudan. BMC research notes. 2014;7(1):531.

20. Gabbad AA, Alrahman GMA, Elawad MA. Childhood pneumonia at omdurman paediatric hospital, Khartoum, Sudan. Int J of Multidisciplinary and Current research. 2014.

21. Ndosa A, Kidenya BR, Mushi MF, Mirambo MM, Hokororo A, Mshana SE. Factors associated with colonization of Streptococcus pneumoniae among under-fives attending clinic in Mwanza City, Tanzania. Tanzania Journal of Health Research. 2015;17(1).

22. Lugangira K, Kalokola F. Morbidity and mortality of children aged 2–59 months admitted in the Tanzania Lake Zone’s public hospitals: a cross-sectional study. BMC research notes. 2017;10(1):502.

23. Lindstrand A, Kalyango J, Alfven T, Darenberg J, Kadobera D, Bwanga F, et al. Pneumococcal carriage in children under five years in Uganda-will present pneumococcal conjugate vaccines be appropriate? PloS one. 2016;11(11).

24. Tuhebwe D, Tumushabe E, Leontsini E, Wanyenze RK. Pneumonia among children under five in Uganda: symptom recognition and actions taken by caretakers. African health sciences. 2014;14(4):993-1000.

25. Negash AA, Asrat D, Abebe W, Hailemariam T, Hailu T, Aseffa A, et al., editors. Bacteremic community-acquired pneumonia in Ethiopian children: etiology, antibiotic resistance, risk factors, and clinical outcome. Open forum infectious diseases; 2019: Oxford University Press US.

26. Ásbjörnsdóttir KH, Slyker JA, Weiss NS, Mbori-Ngacha D, Maleche-Obimbo E, Wamalwa D, et al. Breastfeeding is associated with decreased pneumonia incidence among HIV-exposed, uninfected Kenyan infants. AIDS (London, England). 2013;27(17):2809.

27. Deng AA. Risk factors for acute lower respiratory tract infections in children under five years of age in Juba, Southern Sudan: JKUAT-COHES; 2019.

28. Onyango D, Kikuvi G, Amukoye E, Omolo J. Risk factors of severe pneumonia among children aged 2-59 months in western Kenya: a case control study. Pan African Medical Journal. 2012;13(1).

29. Deribew A, Tessema F, Girma B. Determinants of under-five mortality in Gilgel gibe field research center, Southwest Ethiopia. Ethiopian Journal of Health Development. 2007;21(2):117-24.

30. MANYA AS. RISK FACTORS FOR PNEUMONIA IN CHILDREN UNDER FIVE YEARS OF AGE, HOSPITALIZED IN A RURAL DISTRICT HOSPITAL OF WESTERN KENYA, 2005.

31. Workineh Y, Hailu D, Gultie T. Determinants of pneumonia among under two children in southern Ethiopia: A case control study 2016. Current Pediatric Research. 2017;21(4).

32. Markos Y, Dadi AF, Demisse AG, Ayanaw Habitu Y, Derseh BT, Debalkie G. Determinants of Under-Five Pneumonia at Gondar University Hospital, Northwest Ethiopia: An Unmatched Case-Control Study. Journal of environmental and public health. 2019;2019.

33. Gedefaw M, Berhe R. Determinates of childhood pneumonia and diarrhea with special emphasis to exclusive breastfeeding in north Achefer district, northwest Ethiopia: a case control study. Open Journal of Epidemiology. 2015;5(02):107.
