# Supplementary material for: Prevalence of pneumonia and its associated factors among under-five children in East Africa: a systematic review and meta-analysis
Source: BMC Pediatr. 2020 May 27;20:254. doi: 10.1186/s12887-020-02083-z (PMC7251746; doi:10.1186/s12887-020-02083-z)
Supplement: Supplementary file 3 — Additional file 3 Table S1. Search strategy used for one of the databases [file 12887_2020_2083_MOESM3_ESM.docx]

# Table S1. Search strategy used for one of the databases

| Medline/PubMed | | |  |
| --- | --- | --- | --- |
|  | **Search terms** | |  |
| Group | **Non-MeSH terms** | **MeSH (sub-terms in MeSH)** | **Citations** |
| #1 | Prevalence  Magnitude  Epidemiology |  |  |
| #2 | Causes  Determinants  Associated factors  Predictors  Risk factors |  |  |
| #3 | under five  childhood  Infant  Child  Children | Children |  |
|  |  |  |  |
| #4 | Respiratory tract infection | Pneumonia |  |
| #5 | Eastern Africa |  |  |
| #1 AND #2 AND #3 AND #4 AND #5 |  |  | **2252** |

(Prevalence OR magnitude OR epidemiology) AND (causes OR determinants OR associated factors OR predictors OR risk factors) AND (children [MeSH Terms] OR under five OR child OR childhood) AND (pneumonia [MeSH Terms] OR respiratory tract infection) AND Eastern Africa
