# Supplementary material for: Prevalence of pneumonia and its associated factors among under-five children in East Africa: a systematic review and meta-analysis
Source: BMC Pediatr. 2020 May 27;20:254. doi: 10.1186/s12887-020-02083-z (PMC7251746; doi:10.1186/s12887-020-02083-z)
Supplement: Supplementary file 5 — Additional file 5 Table S3. Adjusted confounders and main findings extracted from included studies in East Africa [file 12887_2020_2083_MOESM5_ESM.docx]

Table S3: Adjusted confounders and main findings extracted from included studies in East Africa

| Author | Adjusted confounders | Main findings |
| --- | --- | --- |
| Lema *et al*(1) | Sex of child, Fuel used for cooking, Place of food cooking, Number of windows in the main house, Types of breast feeding and Diarrhea in the last 2 wks | - The prevalence of community acquired pneumonia to be 17.7%. - The potential factors identified in this study were being male, caring of child on mothers back during food cooking, history of acute respiratory tract infection, and children who were living in the house that have three or more window |
| Tadesse *et al*(2) | Parental smoking, non-vaccination with PCV10, female sex , age, weight-for age z-score (WAZ) <–2 SDs, breastfeeding, cooking fuel and Cooking performed in living rooms | - The prevalence of bacteremic pneumonia was 5.6%. - Staphylococcus aureus (26.5%) was the predominant pathogenic species, followed by Enterococcus faecium (11.8%), Escherichia coli (11.8%), and Klebsiella pneumoniae (11.8%). - The overall in-hospital case fatality rate was 2.37% (13/549), and WAZ <–3 SDs (OR, 13.5; 95% CI, 3.95–46.12) was associated with mortality. |
| Abaye *et al*(3) | Nonexclusive breast feeding,  Children living with siblings ,> 5 person per household, Non- exclusive breast feeding, horse cart transportation increases carriage and URTIs in the last 3 months one or more episode | - Overall pneumococcal carriage were 18.4% (88/477). - Children living with siblings (1–2) < 6 years in household ,> 5 person per household,Non- exclusive breast feeding, horse cart transportation increases carriage. - S. pneumoniae showed 21 (23.9%) resistance to erythromycin, 18 (20.4%) to amoxicillin,13 (15.0%) to penicillin, and the least 1 (1.1%) to augmentin. |
| Fekadu *et al*(4) | Immunization History, Birth order, Stunting, using charcoal for cooking, carrying the child on back during cooking, keeping cattle inside the main house and living in crowded house were variables found associated with pneumonia | - The overall prevalence of pneumonia was 16.1%. - Stunting, using charcoal for cooking, carrying the child on back during cooking, keeping cattle inside the main house and living in crowded house were variables found associated with pneumonia |
| Shibre *et al*(5) |  |  |
| Shah *et al*(6) | Gestation age (weeks), Birth weight (kg) | - Pneumonia exhibited higher mortality rates (13.4 %,). - The highest mortality rate (10.3%) was seen in patient’s admitted <1 h after birth. - birth weight <2 kg (p<0.01), birth weight   between 2.1 and 2.5 kg (p<0.01), Apgar score at  1 min (p<0.01), small for gestational age (p<0.01),  hypothermia (p<0.04) and pneumonia (p<0.01) were  associated with mortality. |
| Tegenu *et al*(7) | Immunization History, Birth order, Stunting, using charcoal for cooking, carrying the child on back during cooking, keeping cattle inside the main house and living in crowded house were variables found associated with pneumonia | - |
| Abuka *et al*(8) | Fuel used for Cooking, Absence of separate kitchen, absence of window in the kitchen, breast feeding, Place of the child during  Cooking and cigarette smoker | - Prevalence of pneumonia was 33.5%. - Absence of separate kitchen, absence of window in the kitchen, breast feeding less than one year and children at age range of 2-12 months were identified determinates. |
| Adhanom *et al* (9) | Young age (18-29), recent CD4+ count less than 350 cells/mL, alcohol consumption, and HIV WHO stage II | 43.7% were positive for various bacterial species. The predominant bacterial species were Klebsiella pneumoniae (n=26, 23.6 %) followed  by Streptococcus pneumoniae (n=17, 15.5 %), Escherichia coli (n=16, 14.5%), Klebsiella spp. (n=15, 13.6%), Staphylococcus aureus  (n=9, 8.2%), Enterobacter spp. (n=7, 6.3%), Pseudomonas aeruginosa (4, n=3.6%), Proteus spp. (n=4, 3.6%), Citrobacter freundii  (n=7, 6.3%), Streptococcus pyogenes (3, 2.7%), and Haemophilus influenzae (n=2, 1.8%). |
| Lenda *et al*(10) | Sex of child, Fuel used for cooking, Place of food cooking, Number of windows in the main house, Types of breast feeding and Diarrhea in the last 2 wks | - |
| Keter *et al* (11) | - | Mud walled house, house without windows, overcrowding, cooking fuel, cooking near to the bed |
| Muthumbi *et al*(12) | HIV infection, anemia, splenomegaly, recent history of pneumonia, history of pneumonia >2 years previously, coryza in the 2 weeks preceding hospitalization, current smoking, use of khat, use of snuff and contact with several animal species. | - Pneumonia was associated with HIV infection (Odds Ratio [OR] 2.06, anemia (OR 1.91), splenomegaly (OR 2.04), recent history of pneumonia (OR 4.65), history of pneumonia >2 years previously (OR 17.13, 95% ), coryza in the 2 weeks preceding hospitalization (OR 2.09), current smoking (2.19, 95% ), use of khat (OR 3.44), use of snuff (OR 2.67) and contact with several animal species. - Presence of a Bacillus Calmette-Guerin (BCG) scar was associated with protection (OR 0.51, 95% CI 0.32–0.82). The risk factors varied significantly by sex. |
| Ndungu *et al*(13) | Poor knowledge of symptoms , long distance to the nearest health facility, means of transport used to access the nearest health facility and previous high expenditure at health facility. Travelling for long distance and long waiting time before service provision during the previous facility visit were the main determinants of delayed facility consultations. | - 74.3% of the children had an episode of ARI in the preceding 2 weeks. - Poor knowledge of pneumonia was documented in 92.5% of caretakers. - 62.1% of caretakers had delayed health facility consultation for ARI. - logistic regression, the main factors that independently determined delayed facility consultation were long waiting time(>1hour) before service provision (p = 0.001), use of a motorbike to access the nearest health facility (p = 0.001) and traveling for 4 - 4.9 kilo meters to the nearest health facility (p = 0.002). |
| Walekhwa *et al*(14) | - | - 20.39% (n=42) were found to becarriers of SP - About 52% (n=22) of the SPn carriers had received the recommended dose of PCV-10, - Almost all (n=41; 19.90% of subjects) isolates contained non-vaccine - Serotypes 28F, 6A, 11A, 3 and 7C were prevalent in both vaccinated and unvaccinated children, |
| Sikolia et al(30) | Mud walled house, house without windows, overcrowding, cooking fuel, cooking near to the bed | About 67.9% of children had ARTI. Mud walled house, house without windows, overcrowding, cooking fuel, cooking near to the bed found to be significant factors |
| Kinyoki *et al*(15) | - | The empirical correlations of the enumeration area  proportion were 0.37, 0.63 and 0.66 for ARI and stunting, diarrhoea and stunting and ARI and  diarrhoea, respectively.  Spatially, the posterior residual effects ranged 0.03–20.98, 0.16–6.37 and 0.08–9.66 for shared component between ARI and stunting, diarrhoea and stunting and ARI and diarrhoea, respectively. |
| Gritly *et al* (16) | Sex of child, Fuel used for cooking, Place of food cooking, Number of windows in the main house, Types of breast feeding and Diarrhea in the last 2 wks | - |
| Salih *et al* (17) | Mud walled house, house without windows, overcrowding, cooking fuel, cooking near to the bed |  |
| Gabbad *et al* (18) | Age of child, family income, family residence, and mother's education | The proportion of pneumonia in children was 20.2%. The disease was more frequent in males rather than females with Odds Ratio (OR) = 2.4123 - 95% and There was statistical association between pneumonia and age of child, family income (X2 = 26.862 - P value = 0.00000147), family residence (Relative Risk (RR) = 2.657, 95% Confidence Interval (CI) = from 1.9391 to 3.6402) and mother's education (X2 = 56.58, P value = 0.000). |
| Ndosa *et al* (19) | Number of children at home, positive HIV status and someone smoking and Children with chronic diseases | Children with chronic diseases and those at school were 3.4 and 4.4 times more at risk to be carriers of S. pneumoniae  The resistance levels of S.pneumoniae to penicillin, co-trimoxazole and erythromycin were 40%, 88.2% and 41.7%, respectively.  However all of the S. pneumoniae isolates were found to be 100% sensitive to ciprofloxacin. |
| Lugangira *et al* (20) | Female sex, malnutrition, level of maternal education, Education status, Employment status and Ever hospitalized | The major leading morbidities include malaria (49%), anemia (37%), diarrhea (27%), pneumonia (22%) and severe acute malnutrition (21%). We found the case fatality of 74 deaths per 1000 under-five admissions. |
| Lindstrand *et al* (21) | Sex of child, Fuel used for cooking, Place of food cooking, Number of windows in the main house, Types of breast feeding and Diarrhea in the last 2 wks | - |
| Tuhebwe *et al* (22) | - | Age of the child, father’s educational status, child cared by housekeeper, Children’s having history of diarrhea and household history of AURTI, Breast feeding history, parental smoking, and crowding status |
| Negash *et al*(23) | Sex of child, Fuel used for cooking, Place of food cooking, Number of windows in the main house, Types of breast feeding and Diarrhea in the last 2 wks | - |
| Ásbjörnsdóttir *et al*(24) | Crowding in the home, maternal HIV viral load at 32 weeks’ gestation) per log10 difference] and being underweight at the previous visit.. | - The incidence of pneumonia was 89.8/100 child-years (95% CI: 80.1-100.8). - Breastfed infants had a 47% lower risk of pneumonia than those who never breastfed [HR=0.53 (0.39-0.73)]. This association was independent of infant growth and maternal viral load. |
| Deng *et al* (25) | Malnutrition, Income of the parents, Anaemia, mud or cow dung floored house, and Family history of smoking | Majority of caregivers of cases and controls were 88.9% (48/54) and 94.4% (51/54) females and the remainder were 11.1% (6/54) and 5.6% (3/54) males respectively. |
| Dadi et al(16) | Age of the child, father’s educational status, child cared by housekeeper, Children’s having history of diarrhea and household history of AURTI, Breast feeding history, parental smoking, and crowding status | - The risk of pneumonia was diminished among children in the 2 - 11 months age group) and 12 - 23 months age group - An increased risk of pneumonia was associated with the father’s primary education ,child cared by housekeeper, Children’s having history of diarrhea and household history of acute lower respiratory infection |
| Geleta et al(14) | Maternal age, history of AURTI, current parental smoking, more than four family members, non- exclusive breast feeding, lack of zinc supplementation, wasting, and  family Size | Maternal age (AOR= 5.3; 95% CI: 1.9, 14.3), previous upper respiratory tract infection (AOR= 5.2; 95% CI: 3.1, 8.9), current parental smoking (AOR= 1.9; 95% CI: 1.1, 3.7), more than four family members (AOR= 2.1; 95% CI: 1.1, 3.9), non- exclusive breast feeding during the first six month of life (AOR= 3.3; 95% CI: 2.0, 5.4), lack of zinc supplementation (AOR= 1.7; 95% CI: 1.1, 2.8) and wasting (AOR= 2.0; 95% CI: 1.2, 3.5) were determinants of Community Acquired Pneumonia among 2-59 months old children. |
| Onyango *et al*(26) | Use wood as fuel source, cook food in living room, caring of the child on mothers back or besides the mother during cooking, absence of windows in the kitchen, children who unvaccinated, Vitamin A supplementation, and moderate acute malnutrition | Prevalence of pneumonia was 28.1%. Use wood as fuel source(P= 0.003, AOR=3.45), cook food in living room(P= 0.008, AOR= 3.34), caring of the child on mothers back or besides the mother during cooking (P= 0.008, AOR= 2.96), absence of windows in the kitchen(P= 0.001, AOR= 2.5), children who unvaccinated(P= 0.004, AOR= 4.6), Vitamin A supplementation(P= 0.002, AOR= 0.168), mixed breast feeding during 6 months(P= 0.014, AOR= 3.26), moderate acute malnutrition(P= 0.002, AOR= 4) and child history of ARTI(P= 0.004, AOR= 4) were a potential determinates of under-five children pneumonia |
| Deribew *et al* (27) | Age of the child, father’s educational status, child cared by housekeeper, Children’s having history of diarrhea and household history of AURTI, Breast feeding history, parental smoking, and crowding status | - |
| MANYA *et al*(28) |  |  |
| Workineh *et al*(29) | Marital status, Maternal educational status, Vitamin A  Utilization, being vaccinated, and exclusive breast feeding | - Ninety seven (17.4%), 245 (43.9%) and 119 (21.3%) of children were underweight, wasted and stunted respectively. - Being married, completing secondary school and above, children without young siblings breastfeeding within an hour of delivery, being vaccinated, and exclusive breastfeeding were protective against pneumonia |
| Markos *et al*(30) | diarrhea in the past  2 weeks, children’s mothers who did not hear about how to handle  domestic smoking , and children of mothers who did not follow proper hand washing practice and absence of window | An increased odds of pneumonia was associated with children who had diarrhea in the past  fifteen days of data collection, children’s mothers who did not hear about how to handle  domestic smoking , and children of mothers who did not follow proper hand washing practice |
| Gedefaw *et al*(31) | Birth weight < 2500, Pre-lacteal feeds given, Exclusive breast-feeding, Maternal education and Monthly income less than 850 | - About 83% of the controls and only 12.3% of the cases were exclusively breast fed. Children who were exclusively breast fed were 83 times less likely to develop pneumonia - Marital status, monthly income, prelacteal feeding, and late initiation of breast feeding were found to have statistically significant association with childhood diarrhea and pneumonia. - This study brought local evidence that exclusive breast feeding had a protective effect against common childhood infectious dis- eases—pneumonia and diarrhoea—in the study area. |
| Markos *et al*(30) | Age of the child, father’s educational status, child cared by housekeeper, Children’s having history of diarrhea and household history of AURTI, Breast feeding history, parental smoking, and crowding status | Age of the child, father’s educational status, child cared by housekeeper, Children’s having history of diarrhea and household history of AURTI, Breast feeding history, parental smoking, and crowding status |

Reference

1. Lema B, Seyoum K, Atlaw D. Prevalence of Community Acquired Pneumonia among Children 2 to 59 Months Old and its Associated Factors in Munesa District, Arsi Zone, Oromia Region, Ethiopia. Clinics Mother Child Health. 2019;16:334.

2. Tadesse R. Household Biomass Fuel Use and Acute Respiratory Infections among Younger Children: An Exposure Assessment in Anilemo Woreda, Southern Ethiopia: Addis Ababa University; 2015.

3. Abaye G, Fekadu H, Haji K, Alemu D, Anjulo AA, Yadate DT. Prevalence and risk factors of pneumococcal nasopharyngeal carriage in healthy children attending kindergarten, in district of Arsi Zone, South East, Ethiopia. BMC research notes. 2019;12(1):253.

4. Fekadu GA, Terefe MW, Alemie GA. Prevalence of pneumonia among under-five children in Este Town and the surrounding rural Kebeles, Northwest Ethiopia: a community based cross sectional study. Science Journal of Public Health. 2014;2(3):150-5.

5. Shibre G. Assessment of the Prevalence and Associated Factors of Pneumonia in Children 2to 59 Months Old, Debreberhan District, North East Ethiopia: Addis Abeba University; 2015.

6. Shah S, Zemichael O, Meng HD. Factors associated with mortality and length of stay in hospitalised neonates in Eritrea, Africa: a cross-sectional study. BMJ open. 2012;2(5):e000792.

7. Tegenu K. Prevalence and associated factors of pneumonia among under-five children at public hospitals in Jimma zone, South West of Ethiopia, 2018: Addis Ababa Universty; 2018.

8. Abuka T. Prevalence of pneumonia and factors associated among children 2-59 months old in Wondo Genet district, Sidama zone, SNNPR, Ethiopia. Current Pediatric Research. 2017.

9. Adhanom G, Gebreegziabiher D, Weldu Y, Gebreyesus Wasihun A, Araya T, Legese H, et al. Species, Risk Factors, and Antimicrobial Susceptibility Profiles of Bacterial Isolates from HIV-Infected Patients Suspected to Have Pneumonia in Mekelle Zone, Tigray, Northern Ethiopia. BioMed research international. 2019;2019.

10. Lenda A, Demena M, Mengistie B. Prevalence of Pneumonia and Associated Factors Among Under-five Children in Boloso Bombe Woreda, Southern Ethiopia: A Community Based Study: Haramaya University; 2018.

11. Keter PKK. Knowledge, Attitudes and Practices of Mothers in relation to Childhood Pneumonia and factors associated with Pneumonia and Seeking Health Care in Kapsabet District Hospital in Nandi County, Kenya: JKUAT; 2015.

12. Muthumbi E, Lowe BS, Muyodi C, Getambu E, Gleeson F, Scott JAG. Risk factors for community-acquired pneumonia among adults in Kenya: a case–control study. Pneumonia. 2017;9(1):17.

13. Ndungu EW, Okwara FN, Oyore JP. Cross Sectional Survey of Care Seeking For Acute Respiratory Illness in Children Under 5 Years in Rural Kenya. Am J Pediatr. 2018;4(3):69-79.

14. Walekhwa M, Muturi M, Revathi Gunturu EK, Kabera B. Streptococcus pneumoniae serotype epidemiology among PCV-10 vaccinated and unvaccinated children at Gertrude’s Children’s Hospital, Nairobi County: a cross-sectional study. F1000Research. 2018;7.

15. Kinyoki DK, Manda SO, Moloney GM, Odundo EO, Berkley JA, Noor AM, et al. Modelling the ecological comorbidity of acute respiratory infection, diarrhoea and stunting among children under the age of 5 years in Somalia. International Statistical Review. 2017;85(1):164-76.

16. Gritly SM, Elamin MO, Rahimtullah H, Ali AYH, Dhiblaw A, Mohamed EA, et al. Risk factors of pneumonia among children under 5 years at a pediatric hospital in Sudan. International Journal of Medical Research & Health Sciences. 2018;7(4):60-8.

17. Salih KEM, Bilal JA, Alfadeel MA, Hamid Y, Eldouch W, Elsammani E, et al. Poor adherence to the World Health Organization guidelines of treatment of severe pneumonia in children at Khartoum, Sudan. BMC research notes. 2014;7(1):531.

18. Gabbad AA, Alrahman GMA, Elawad MA. Childhood pneumonia at omdurman paediatric hospital, Khartoum, Sudan. Int J of Multidisciplinary and Current research. 2014.

19. Ndosa A, Kidenya BR, Mushi MF, Mirambo MM, Hokororo A, Mshana SE. Factors associated with colonization of Streptococcus pneumoniae among under-fives attending clinic in Mwanza City, Tanzania. Tanzania Journal of Health Research. 2015;17(1).

20. Lugangira K, Kalokola F. Morbidity and mortality of children aged 2–59 months admitted in the Tanzania Lake Zone’s public hospitals: a cross-sectional study. BMC research notes. 2017;10(1):502.

21. Lindstrand A, Kalyango J, Alfven T, Darenberg J, Kadobera D, Bwanga F, et al. Pneumococcal carriage in children under five years in Uganda-will present pneumococcal conjugate vaccines be appropriate? PloS one. 2016;11(11).

22. Tuhebwe D, Tumushabe E, Leontsini E, Wanyenze RK. Pneumonia among children under five in Uganda: symptom recognition and actions taken by caretakers. African health sciences. 2014;14(4):993-1000.

23. Negash AA, Asrat D, Abebe W, Hailemariam T, Hailu T, Aseffa A, et al., editors. Bacteremic community-acquired pneumonia in Ethiopian children: etiology, antibiotic resistance, risk factors, and clinical outcome. Open forum infectious diseases; 2019: Oxford University Press US.

24. Ásbjörnsdóttir KH, Slyker JA, Weiss NS, Mbori-Ngacha D, Maleche-Obimbo E, Wamalwa D, et al. Breastfeeding is associated with decreased pneumonia incidence among HIV-exposed, uninfected Kenyan infants. AIDS (London, England). 2013;27(17):2809.

25. Deng AA. Risk factors for acute lower respiratory tract infections in children under five years of age in Juba, Southern Sudan: JKUAT-COHES; 2019.

26. Onyango D, Kikuvi G, Amukoye E, Omolo J. Risk factors of severe pneumonia among children aged 2-59 months in western Kenya: a case control study. Pan African Medical Journal. 2012;13(1).

27. Deribew A, Tessema F, Girma B. Determinants of under-five mortality in Gilgel gibe field research center, Southwest Ethiopia. Ethiopian Journal of Health Development. 2007;21(2):117-24.

28. MANYA AS. RISK FACTORS FOR PNEUMONIA IN CHILDREN UNDER FIVE YEARS OF AGE, HOSPITALIZED IN A RURAL DISTRICT HOSPITAL OF WESTERN KENYA, 2005.

29. Workineh Y, Hailu D, Gultie T. Determinants of pneumonia among under two children in southern Ethiopia: A case control study 2016. Current Pediatric Research. 2017;21(4).

30. Markos Y, Dadi AF, Demisse AG, Ayanaw Habitu Y, Derseh BT, Debalkie G. Determinants of Under-Five Pneumonia at Gondar University Hospital, Northwest Ethiopia: An Unmatched Case-Control Study. Journal of environmental and public health. 2019;2019.

31. Gedefaw M, Berhe R. Determinates of childhood pneumonia and diarrhea with special emphasis to exclusive breastfeeding in north Achefer district, northwest Ethiopia: a case control study. Open Journal of Epidemiology. 2015;5(02):107.
